# The power of GM-CSF: immune regulation in the defense against *Phialophora verrucosa* infection

**DOI:** 10.3389/fimmu.2025.1662183

**Published:** 2025-10-20

**Authors:** Qi Dong, Jiejie Lu, Mengying Liu, Weiwei Wu, Yuying Kang, Ruijun Zhang

**Affiliations:** ^1^ Department of Dermatology, Third Hospital of Shanxi Medical University, Shanxi Bethune Hospital, Shanxi Academy of Medical Sciences, Tongji Shanxi Hospital, Taiyuan, China; ^2^ Department of Dermatology, Affiliated Dermatology Hospital of Hainan Medical University, Haikou, China; ^3^ Department of Dermatology, the Fifth People’s Hospital of Hainan, Haikou, China

**Keywords:** GM-CSF, *Phialophora verrucosa*, immune response, neutrophils, macrophages

## Abstract

**Background:**

*Phialophora verrucosa*, a dematiaceous fungus, causes serious infections such as phaeohyphomycosis. These infections can severely impair patient quality of life and may be life-threatening. Current understanding of host immune defenses against this pathogen remains limited.

**Objective:**

This study aims to investigate the role of granulocyte-macrophage colony-stimulating factor (GM-CSF) in host defense against *P. verrucosa*. Using both *in vivo* and *in vitro* models, the current study specifically examines how GM-CSF deficiency impacts immune responses and fungal clearance.

**Methods:**

C57BL/6 wild-type (WT) and GM-CSF-deficient (*Csf2* KO) mice were infected subcutaneously in the footpad with live *P. verrucosa* conidia. Skin lesion appearance and foot swelling were monitored for 4 weeks. At specific time points, footpad tissues were collected for PAS, CD68, and MPO staining, and colony-forming units were calculated to assess fungal load. Serum and tissue homogenates were analyzed for cytokine levels. Bone marrow-derived macrophages and neutrophils were isolated to evaluate GM-CSF’ s impact on chemotaxis, phagocytosis, and killing functions.

**Results:**

Compared to WT mice, GM-CSF deficiency significantly delayed fungal clearance and prolonged disease progression, accompanied by reduced inflammatory responses and decreased neutrophil infiltration. *In vitro*, GM-CSF supplementation restored macrophage chemotaxis and enhanced neutrophil phagocytic activity, but did not affect their killing efficiency. A compensatory increase in IFN-γ levels in *Csf2* KO mice was insufficient to overcome the immune defects caused by GM-CSF deficiency.

**Conclusion:**

This study reveals the indispensable role of GM-CSF in antifungal immunity and its potential as a therapeutic target for controlling infections caused by dematiaceous fungi.

## 1 Introduction

Dematiaceous fungi, characterized by their natural brown or melanin content, can cause chromoblastomycosis, phaeohyphomycosis, or mycetoma (1). Phaeohyphomycosis, once considered a rare infection, has been increasingly reported worldwide in recent decades, particularly in tropical and subtropical regions (2). This trend is attributed to factors such as immunosuppressive therapies, advanced diagnostic techniques, and greater clinical awareness. In China, the incidence of phaeohyphomycosis has also shown a significant upward trajectory, mirroring the global pattern and posing a growing clinical challenge (3). This infection primarily affects the skin, resulting in persistent, hard-to-treat lesions that can progress aggressively, severely impacting patients’ quality of life and even posing life-threatening risks. However, the mechanisms underlying the interaction between these infections and the host remain unclear. Recent studies have highlighted a strong association between caspase recruitment domain-containing protein 9 (CARD9) deficiency and susceptibility to dematiaceous fungal infections, particularly phaeohyphomycosis (4). CARD9 plays a crucial role in regulating the expression of granulocyte-macrophage colony-stimulating factor (GM-CSF) (5). GM-CSF is a pleiotropic cytokine produced by a variety of immune cells, including T lymphocytes, macrophages, and dendritic cells, that functions both as a hematopoietic growth factor and an immune modulator (6, 7). Research indicates that CARD9 deficiency leads to impaired cytokine secretion in both innate and adaptive immune responses, with a significant reduction in GM-CSF expression, thereby compromising host immunity and increasing susceptibility to dematiaceous fungal infections (6). These findings suggest that GM-CSF may play a pivotal role in defending against such infections.

GM-CSF, encoded by the colony-stimulating factor 2 (*Csf2*) gene, regulates the proliferation, differentiation, and maturation of myeloid cells such as granulocytes and macrophages, and it directly or indirectly influences T lymphocyte proliferation, differentiation, and function, thereby bridging innate and adaptive immunity (7). Recent studies have demonstrated that GM-CSF enhances the anti-fungal activity of neutrophils and monocytes against various fungal pathogens, including *Candida albicans*, *Aspergillus fumigatus*, *Penicillium marneffei*, and *Cryptococcus neoformans* (8–10). Clinical research has also highlighted the potential therapeutic efficacy of GM-CSF as an adjunctive treatments for invasive fungal infections caused by *Candida*, *Aspergillus*, and *Mucor* species (11). Despite these advances, the mechanisms underlying GM-CSF’s role in host defense against dematiaceous fungal infections remain poorly understood. Furthermore, while its role is established for common fungi like *Candida* and *Aspergillus*, its specific functions in combating dematiaceous fungi, particularly through regulation of innate immune cells such as neutrophils and macrophages, are largely unexplored and represent a critical gap in knowledge (12). Therefore, this study aims to investigate the mechanisms by which GM-CSF contributes to immune responses against dematiaceous fungal infections, providing new insights and potential therapeutic evidence.

Given the increasing prevalence of *Phialophora verrucosa* (*P. verrucosa*), a major fungal pathogen in China (13, 14), this study has selected this species as a representative dematiaceous fungus to establish both subcutaneous infection models and *in vitro* functional assays using murine immune cells. Through these experimental observations, this study aims to explore the role of GM-CSF in the pathogenesis of infections caused by *P. verrucosa*.

## 2 Materials and methods

### 2.1 Ethics statement

Animal experiments with *P. verrucosa* required an Animal Biosafety Level 2 (ABSL-2) laboratory, as specified by the “Directory of Pathogenic Microorganisms Infectious to Humans” from China’s National Health Commission. Since our institution lacked ABSL-2 capabilities, all experiments were performed at Shanghai Yishang Biotechnology Co., Ltd., which provides certified ABSL-2 facilities. The animal study protocol was reviewed and approved by the Ethics Committee of Shanghai Yishang Biotechnology Co., Ltd. (protocol code IACUC-2023-Mi-203).

### 2.2 Animals and conidia preparation

**Figure 1:**
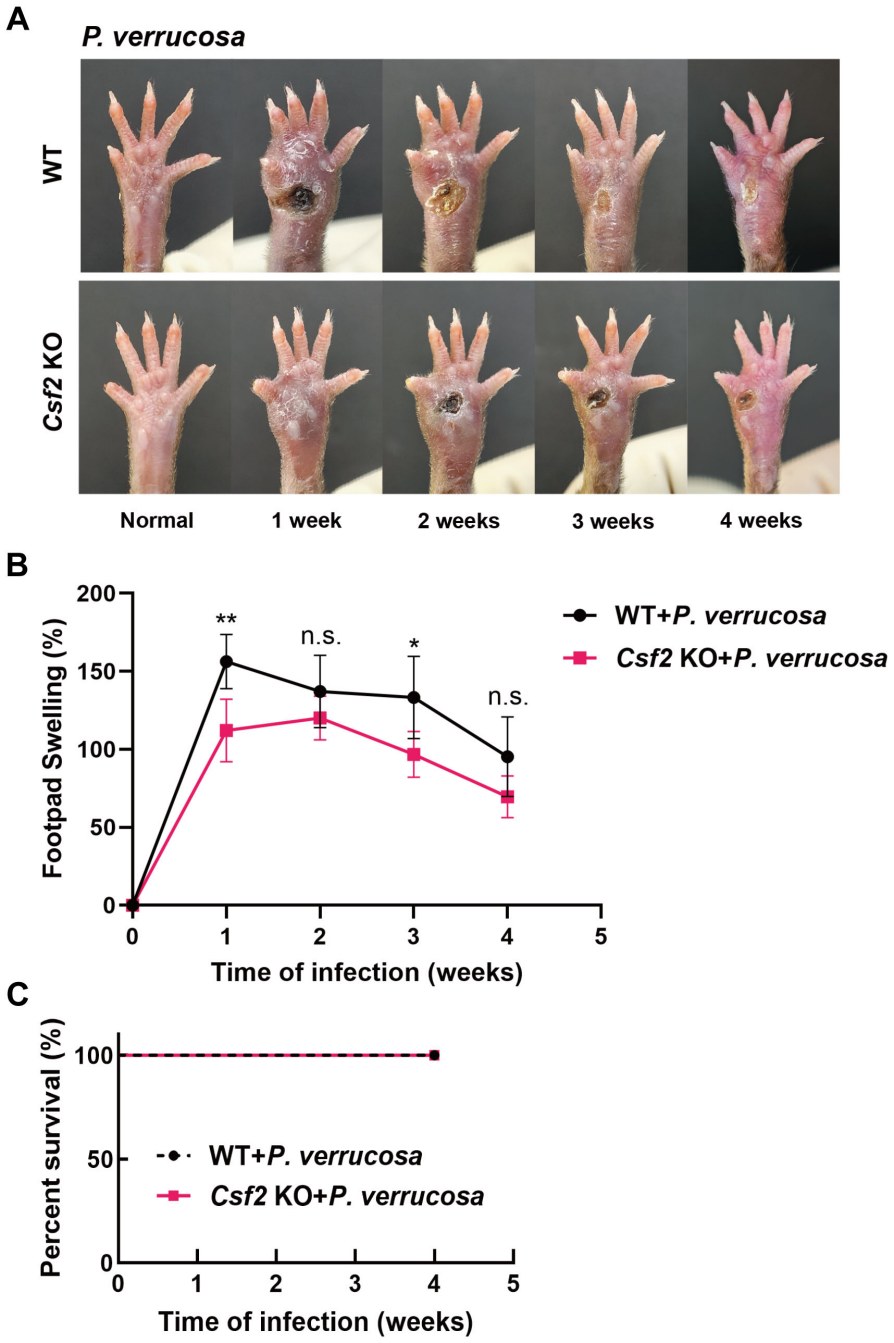
Disease progression in WT and *Csf2* KO mice following subcutaneous *P. verrucosa* infection. WT mice (n=5) and *Csf2* KO mice (n=5) were subcutaneously inoculated with 5×10^7^ live conidia of *P. verrucosa* and observed continuously for four weeks. **(A)** Skin lesion development over time. **(B)** Measurement of footpad swelling. **(C)** Mice survival rates. Data are representative of two independent experiments. *P<0.05, **P<0.01. n.s. indicates no significant difference.

Throughout the study, male and female mice on a C57BL/6J background, aged 6 to 12 weeks were used. Mouse lines included, wild-type (WT) mice (Shanghai Biokai Ke Yi Biotechnology Co., Ltd.), and *Csf2*-knockout (KO) mice (Shanghai Model Organisms Center, Inc., Cat. NO. NM-KO-190532). Two independent replicate experiments were performed, each including 25 mice per group (WT and *Csf*2 KO; 12 females, 13 males) with a verified pure C57BL/6J genetic background (see **Supplementary Files 1**, **2**). Tissue samples for histopathology were collected at six time points (Week 0, Day 3, Weeks 1–4), with Day 3 samples reserved for immunohistochemistry (IHC) to capture early immune cell infiltration (analyzed at Day 0, 3, 7, and 14). For fungal burden and cytokine assays, samples were collected at five core time points (Week 0, Weeks 1–4), with 3–4 biological replicates per time point. Additional mice from the longitudinal survival cohort (100% survival, **Figure 1**) were included to supplement Week 4 samples. All mice were co-housed in a standard Specific Pathogen-Free animal facility at Shanghai Yishang Biotechnology Co., Ltd., under identical conditions. They were provided with autoclaved bedding, food, and water, and all handling was performed within a biosafety cabinet.

The *P. verrucosa* strain was provided by Professor Ruoyu Li from Peking University First Hospital. To prepare the fungal inocula, *P. verrucosa* isolates were cultured on potato dextrose agar (PDA, Solarbio) at 28 °C for 2–3 weeks. After washing the solid medium with phosphate-buffered saline (PBS, Servicebio), the fungal suspension was collected and filtered through multiple layers of sterile gauze. Conidia were adjusted to 5×10^8^/mL and heat-inactivated at 100 °C for 30 minutes, and fluorescein isothiocyanate (FITC, Sigma-Aldrich) was used for labeling.

### 2.3 Murine infection model and determination of fungal burden

Groups of WT or *Csf2* KO mice (n=5 per group) were anesthetized by isoflurane (Ruiwode) inhalation and received a subcutaneous injection of 100 μL of a live conidia suspension (5×10^8^/ml *P. verrucosa*) into the subcutaneous tissue of both hind footpads. Following infection, the natural disease progression (lesion appearance) and the degree of footpad swelling were monitored and measured weekly with a digital caliper for a duration of four weeks.

To quantify the fungal load, at designated time points post-infection (weeks 1, 2, 3, and 4), the full-thickness footpad tissue at the injection site (epidermis, dermis, and subcutaneous tissue, excluding fascia and deeper structures) was aseptically excised (n=3 per group per time point), regardless of the presence or absence of a visible lesion, to ensure consistent sampling of the same anatomical region across all mice. The excised tissues were weighed and transferred to a homogenizer. Each sample was homogenized in 500 μL of ice-cold PBS to prepare a tissue homogenate on ice. Serial 10-fold dilutions of the homogenate were prepared using PBS, and 100 μL aliquots of each dilution were spread evenly onto PDA plates. The plates were incubated at 28 °C for 7 days, after which the colony-forming units (CFUs) were counted. The fungal burden was calculated and expressed as Log_10_ CFU per gram of tissue (Log_10_CFU/g).

### 2.4 Histopathological and immunohistochemical analysis

Footpad tissues from the injection site (as described in section 2.3) were harvested weekly fixed in 4% formaldehyde solution for at least 24 hours. Following routine dehydration and embedding in paraffin, tissue sections (4 ;μm thickness) were prepared. The prepared sections were stained with Periodic Acid-Schiff (PAS, Aidisheng) for histopathological examination.

For immunohistochemical analysis, tissue sections were incubated with primary antibodies against the macrophage marker CD68 (Servicebio) and neutrophil marker myeloperoxidase (MPO, Abcam). Antigen retrieval was performed appropriately, followed by incubation with corresponding secondary antibodies and development with diaminobenzidine. For each sample, three random fields of view were captured under consistent background lighting conditions. The percentage area of positive staining (%AREA) for CD68 and MPO was quantitatively analyzed using Image-Pro Plus 6.0 software.

### 2.5 Cytokine protein determination

Under aseptic conditions, footpad tissues from the injection site (n=3) were excised and weighed. For the preparation of tissue lysates, tissues were homogenized in 500 μL of pre-cooled PBS using a tissue grinder on ice. The homogenates were centrifuged at 3,000 rpm (6.3 cm rotor radius) for 15 minutes at 4 °C, and the supernatants were collected and aliquoted for storage. Mice whole blood (n=4) were obtained via retro-orbital bleeding, kept at room temperature for 2 hours to clot, then centrifuged to collect and aliquot the serum for storage. Protein standards were prepared according to the manufacturer’s instructions for the RayPlex MagPro Mouse Inflammation Bead Array 1 (RayBiotech) Standard curves for each cytokine were generated using these standards. For cytokine detection, 25 μL of the prepared serum or tissue supernatant was mixed with 25 μL of RayPlex Multiplex Bead Cocktail and incubated at room temperature with shaking at 1,000 rpm for 2 hours. The beads were washed twice with 200 μL of 1× wash buffer. Next, 50 μL of 1× Streptavidin-PE was added, and the mixture was incubated on an orbital shaker at 1,000 rpm for 30 minutes at room temperature. After washing and resuspending in 150 μL of 1× wash buffer, the samples were analyzed by flow cytometry.

### 2.6 Isolation and culture of bone marrow-derived macrophages and neutrophils

WT or *Csf2* KO mice were euthanized by cervical dislocation. Tibiae and femurs were aseptically excised, and both epiphyses were removed. Bone marrow cells were flushed out using RPMI 1640 medium (Pricella) through a 70 μm cell strainer to obtain a single-cell suspension.

Bone marrow-derived macrophages (BMDMs) were differentiated from bone marrow cells by culturing them in RPMI 1640 complete medium supplemented with 40 ng/mL M-CSF (Peprotech) for five days. The resulting adherent cells were harvested, and macrophage purity was verified by flow cytometry to be >90%. The gating strategy for BMDMs was performed by first selecting single cells based on FSC-A and SSC-A parameters, followed by identification of macrophages as CD11b^+^F4/80^+^ cells. PE/Cyanine7 anti-mouse CD11b Antibody (BioLegend) and Brilliant Violet 421™ anti-mouse F4/80 Antibody (BioLegend) were used for staining.

Neutrophils were isolated using the Neutrophil Isolation Kit (Solarbio) according to the manufacturer’s instructions. The cells were centrifuged at 250 g for 25 minutes at room temperature, and the intermediate fluffy layer containing neutrophils was collected. The purity of the isolated neutrophils was confirmed by flow cytometry to be >80%. The gating strategy was as follows: single cells were first gated based on FSC-A and SSC-A to exclude debris. Subsequently, neutrophils were identified as CD45^+^CD11b^+^Ly-6G^+^ cells within the granulocyte population. The following monoclonal antibodies (mAbs) were used: PerCP/Cyanine5.5 anti-mouse CD45 Recombinant Antibody (BioLegend), PE/Cyanine7 anti-mouse CD11b Antibody (BioLegend), and PE anti-mouse Ly-6G Antibody (BioLegend).

### 2.7 Functional assays of BMDMs and neutrophils

To evaluate the functional responses of immune cells to *P. verrucosa*, a series of *in vitro* assays were performed. For polarization assessment, BMDMs were co-cultured with heat-killed *P. verrucosa* conidia (BMDMs:conidia=1:10) for 48 hours. After incubation, BMDMs were collected into flow cytometry tubes and stained with the following mAbs (BioLegend): APC anti-mouse/human CD11b Antibody, FITC anti-mouse F4/80 Antibody, Brilliant Violet 421™ anti-mouse CD86 Antibody, and PE anti-mouse CD206 (MMR) Antibody. The expression levels of CD86 (M1 marker) and CD206 (M2 marker) on BMDMs were analyzed by flow cytometry to determine the polarization status.

Conidial phagocytosis was measured using FITC-labeled heat-killed conidia. BMDMs or neutrophils from *Csf2* KO mice were divided into three groups and pre-incubated for 1 hour with recombinant mouse GM-CSF (rmGM-CSF, Peprotech) at concentrations of 0 ng/mL, 5 ng/mL, and 10 ng/mL, respectively. FITC-labeled heat-killed *P. verrucosa* conidia were co-incubated with 1×10^6^ BMDMs or neutrophils (cells:conidia=1:1) at 37°C and 5% CO_2_ incubator. After incubation (2 hours for BMDMs, 30 minutes for neutrophils), cells were collected and stained with appropriate surface markers (CD11b and F4/80 for BMDMs; CD45, CD11b, and Ly-6G for neutrophils). The phagocytic activity of BMDMs and neutrophils was assessed by flow cytometry, and quantified as the percentage of FITC-positive cells within the respective gated cell populations.

Conidial killing was measured by co-culturing live *P. verrucosa* conidia with 1×10^5^ BMDMs or neutrophils (cells:conidia=1:5) in a 37°C incubator with 5% CO_2_. Neutrophils were co-incubated for 30 minutes, while macrophages were co-incubated for 2 hours. At the respective time points, 0.5 mL of 1% Triton X-100 (Solarbio) was added to lyse the cells. Subsequently, 50 μL of the samples was diluted 1,000-fold in PBS and plated on PDA. After incubating for 7 days at 28°C, the numbers of CFUs were counted. The percentage of killing was calculated using the formula: sporicidal efficiency (%) = [1 - (CFU at 30 or 120 min/CFU at 0 min)] ×100%.

The chemotaxis assay was conducted using Transwell inserts with 8 μm (for BMDMs) or 3 μm (for neutrophils) polycarbonate membranes (NEST). A total of 1×10^5^ BMDMs or neutrophils were added to the upper chamber in 400 μL volume. The lower chamber contained heat-killed *P. verrucosa* conidia suspension (cells:conidia=1:10) and RPMI 1640 complete medium, with or without rmGM-CSF, totaling 600 μL per well. The setup was incubated at 37 °C in a 5% CO2 incubator for 12 hours for BMDMs and 30 minutes for neutrophils. Migrated cells were fixed with 4% paraformaldehyde (Servicebio), stained with 1% crystal violet (Bkmamlab). Cells were visualized under an inverted microscope at 250× magnification, and five random fields were photographed and counted.

### 2.8 Statistical analysis

Statistical analysis and graphing were performed using GraphPad Prism 9. Data are presented as mean ± SD. For *in vivo* comparisons across multiple time points, differences between WT and *Csf2* KO mice were analyzed by two-way ANOVA, with a significance threshold of *P* < 0.05. For direct pairwise comparisons *in vitro*, a t-test was applied to assess differences between the two groups, with *P* < 0.05 considered statistically significant.

## 3 Results

### 3.1 Establishment of animal models for subcutaneous infections by *P. verrucosa*

WT and *Csf2* KO mice were subcutaneously inoculated with 5×10^7^ live conidia of *P. verrucosa* and observed for four consecutive weeks. Both groups developed swelling, ulceration, and crusting. WT mice began to show ulceration in the first week, while *Csf2* KO mice exhibited ulceration starting from the second week (**Figure 1A**). Thereafter, both groups showed a tendency towards self-healing, with footpad appearance gradually returning to normal. However, the recovery process was slower in *Csf2* KO mice. Footpad swelling measurements indicated that both WT and *Csf2* KO mice reached peak swelling levels during the early stage of infection (weeks 1-2), followed by a gradual decline (**Figure 1B**). Notably, the overall footpad swelling rate in *Csf2* KO mice was lower than that in WT mice. By the end of the observation period (4 weeks post-infection), all mice in both groups survived, achieving a 100% survival rate (**Figure 1C**).

### 3.2 Deficiency of GM-CSF impairs the mice’s ability to clear *P. verrucosa*

**Figure 2:**
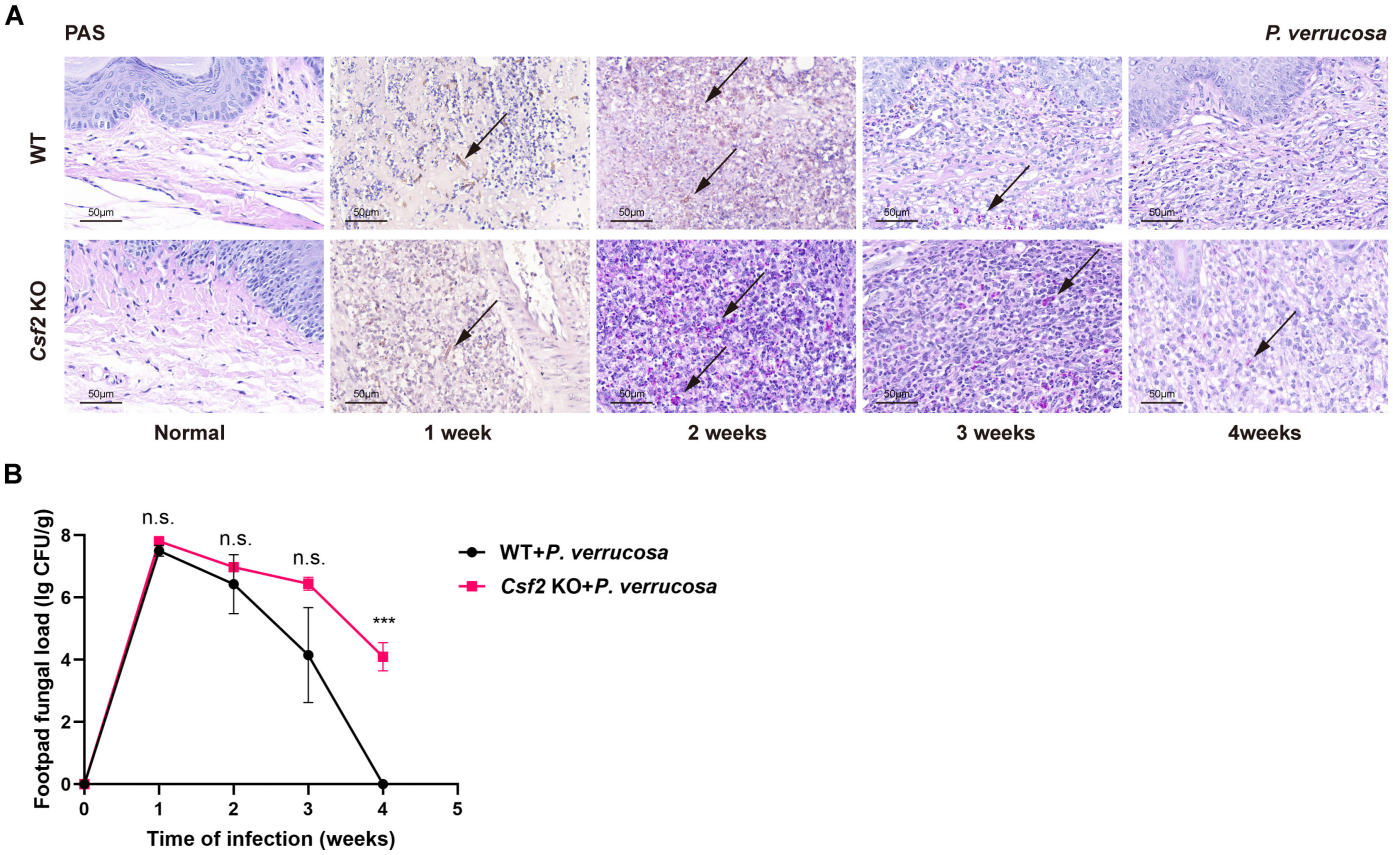
Histopathological features and fungal load in footpad tissue following subcutaneous infection with *P. verrucosa.* **(A)** Histopathological analysis of normal footpads and footpad lesions at 1, 2, 3, 4 weeks post-infection using PAS staining, showing PAS-positive fungal conidia (indicated by arrows). **(B)** Fungal load in harvested footpad tissue (n=3) at different time points following subcutaneous infection with *P. verrucosa* in mice. Data are representative of two independent experiments. ***P<0.001. n.s. indicates no significant difference.

Histopathological examination of footpad tissues showed PAS-positive fungal hyphae in both WT and *Csf2* KO mice at 1 week post-infection. During weeks 2 to 3, only PAS-positive conidia were observed in both groups. By week 4, PAS-positive conidia persisted in *Csf2* KO mice but had disappeared in WT mice (**Figure 2A**). Similarly, fungal load measurements in the harvested footpad tissues revealed that *Csf2* KO mice had a peak fungal load at week 1, followed by slow clearance. By week 4, significant amounts of *P. verrucosa* remained in the footpad tissues of *Csf2* KO mice compared to WT mice, indicating that GM-CSF deficiency delays fungal clearance and prolongs the disease course (**Figure 2B**).

### 3.3 Immune cell infiltration and cytokine levels in *P. verrucosa* infected mice

**Figure 3:**
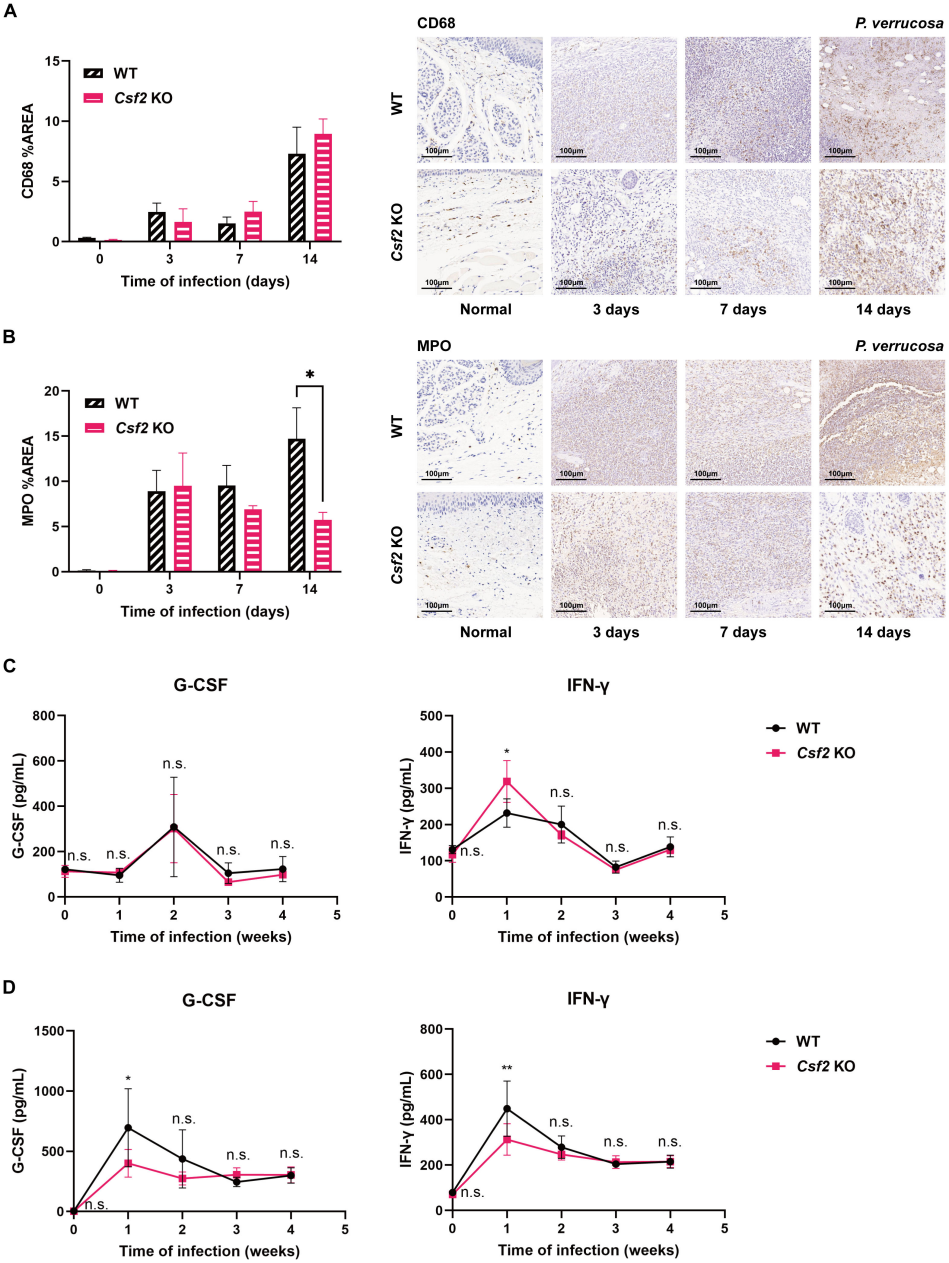
Immune cell infiltration and cytokine levels in WT and *Csf2* KO mice during *P. verrucosa* infection. Immunohistochemical staining of macrophage marker CD68 **(A)** and neutrophil marker myeloperoxidase (MPO) **(B)** in footpad tissues from the injection site of mice infected with *P. verrucosa* for up to 2 weeks. *P < 0.05. WT mice and *Csf2* KO mice were subcutaneously inoculated with 5×10^7^ live conidia in both hind footpads. Cytokine levels in footpad tissue homogenates (n=3) **(C)** and serum (n=4) **(D)** were measured by flow cytometric bead array at weeks 0, 1, 2, 3, and 4 post-infection. Data are representative of two independent experiments. *P < 0.05, **P < 0.01; n.s. indicates no significant difference.

Immunohistochemical staining of the harvested footpad tissues from the injection site of mice infected with *P. verrucosa* for up to 2 weeks showed no significant difference in the percentage area stained for the macrophage marker CD68 between WT and *Csf2* KO mice (**Figure 3A**). However, at 14 days post-infection, the percentage area stained for MPO was significantly higher in WT mice compared to *Csf2* KO mice (**Figure 3B**).

In homogenates of the harvested footpad tissues, IFN-γ levels peaked at week 1 in both groups but remained significantly higher in *Csf2* KO mice compared to WT mice. There was no significant difference in G-CSF levels between the two groups (**Figure 3C**). Serum IFN-γ levels also peaked at week 1, but unlike in tissue homogenates, were significantly higher in WT mice than in *Csf2* KO mice (**Figure 3D**). Similarly, serum G-CSF levels peaked at week 1, with WT mice showing higher levels than *Csf2* KO mice, but no significant differences were observed between the groups thereafter (**Figure 3D**). In both tissue homogenates and serum, there were no significant differences in the expression levels of IL-1β, IL-2, IL-4, IL-6, IL-10, IL-12p70, IL-17, IL-23p19, CXCL1, TNF-α, and MCP-1 between the two groups.

### 3.4 GM-CSF enhances BMDMs chemotaxis but does not affect killing, phagocytosis, or polarization

**Figure 4:**
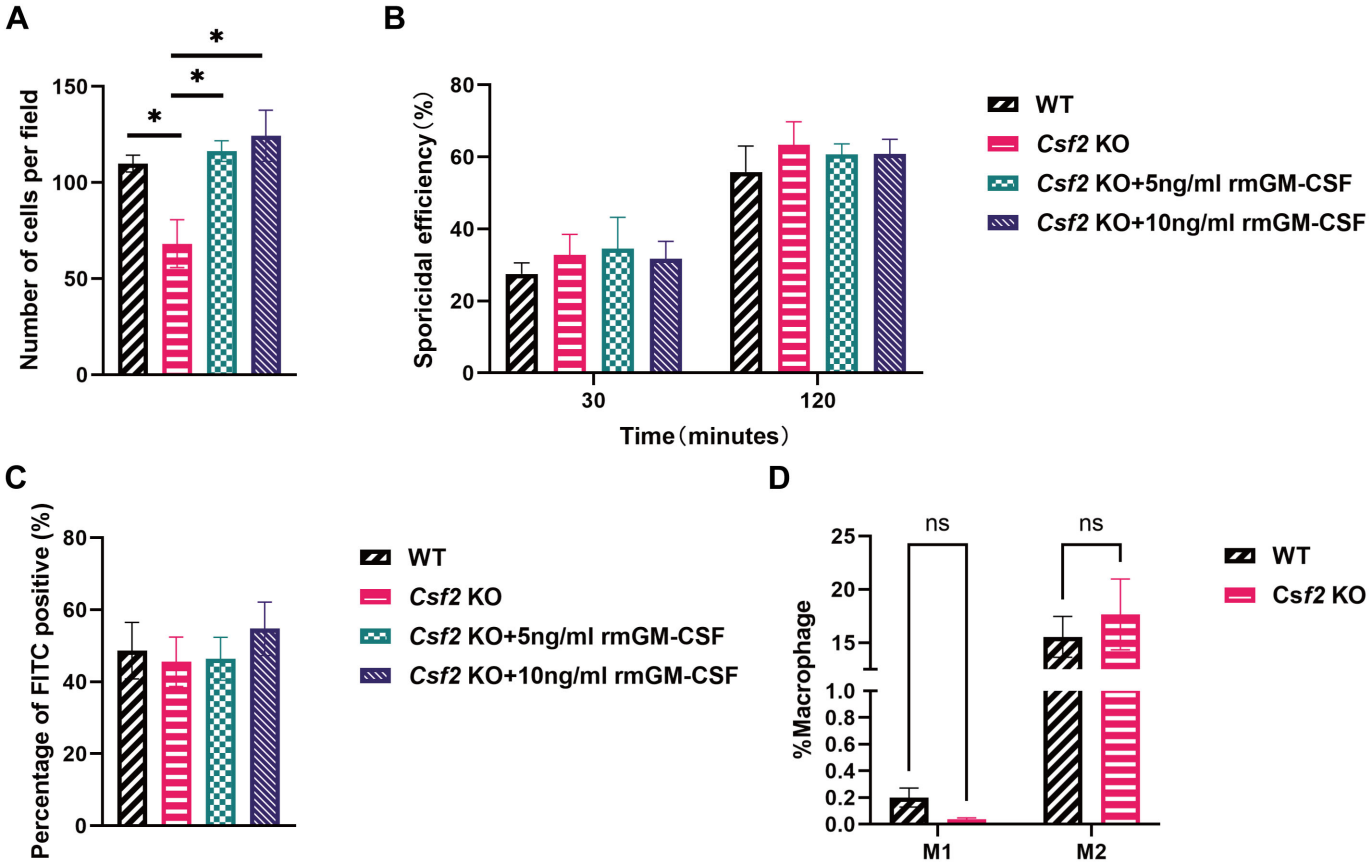
Functional assessment of BMDMs from mice of different genotypes. BMDMs from WT and *Csf2* KO mice were assessed for their interaction with *P. verrucosa* conidia. **(A)** Chemotaxis of macrophages towards conidia (conidia:cells = 10:1) was measured using a transwell chamber after 12 hours of co-incubation, *P < 0.05. **(B)** Killing efficiency of BMDMs against conidia (conidia:cells = 5:1) was evaluated by serial dilution plating following 30 min or 120 min of co-incubation. **(C)** FITC positivity in BMDMs (conidia:cells = 1:1) was determined by flow cytometry after 2 hours of co-incubation. **(D)** BMDMs polarization (conidia:cells = 10:1) was analyzed by flow cytometry following 48 hours of co-incubation. Data are representative of three independent experiments. ns indicates no significant difference.

To assess the chemotaxis ability of BMDMs towards *P. verrucosa* conidia, experiments were conducted using BMDMs from both WT and *Csf2* KO mice. As shown in **Figure 4A**, after a 12-hour incubation at 37 °C, the number of BMDMs migrating towards conidia was significantly higher in WT mice compared to *Csf2* KO mice. Specifically, WT mice exhibited approximately 110 cells per field, while *Csf2* KO mice showed only around 60 cells per field (*P* < 0.05). This observation indicates that the absence of GM-CSF markedly impairs the chemotactic response of macrophages towards conidia. Furthermore, this study found that pre-incubating *Csf2* KO mice’s BMDMs with 5 ng/mL and 10 ng/mL rmGM-CSF could correct the observed chemotactic deficiency. This finding further confirms the critical role of GM-CSF in the chemotactic response of BMDMs to *P. verrucosa* conidia.

The killing efficiency of BMDMs against conidia was evaluated at two time points: 30 minutes and 120 minutes (**Figure 4B**). These results demonstrated that the killing efficiency increased over time for all groups but did not differ significantly between WT and *Csf2* KO mice. At 30 minutes, both WT and *Csf2* KO mice showed approximately 30% killing efficiency. By 120 minutes, this efficiency rose to about 60% for both genotypes, suggesting that GM-CSF deficiency does not substantially affect the macrophage’s capacity to kill *P. verrucosa* conidia. Additionally, supplementation with GM-CSF did not enhance the conidial killing ability of BMDMs.

The percentage of FITC-positive BMDMs was also examined after 2 hours (**Figure 4C**). The results indicated no significant differences among WT and *Csf2* KO mice, with both groups showing similar levels of FITC positivity (approximately 45%). Additionally, supplementation with GM-CSF did not enhance the FITC positivity levels. These findings suggest that GM-CSF deficiency neither impairs nor enhances the phagocytic capacity of BMDMs.

Finally, the polarization state of BMDMs was analyzed after a 48-hour incubation period (**Figure 4D**). The percentages of M1 and M2 polarized macrophages were comparable across WT and *Csf2* KO mice. These findings indicate that GM-CSF deficiency does not significantly alter the polarization profile of macrophages under experimental conditions.

### 3.5 GM-CSF enhances neutrophil phagocytosis, but not chemotaxis or killing ability

**Figure 5:**
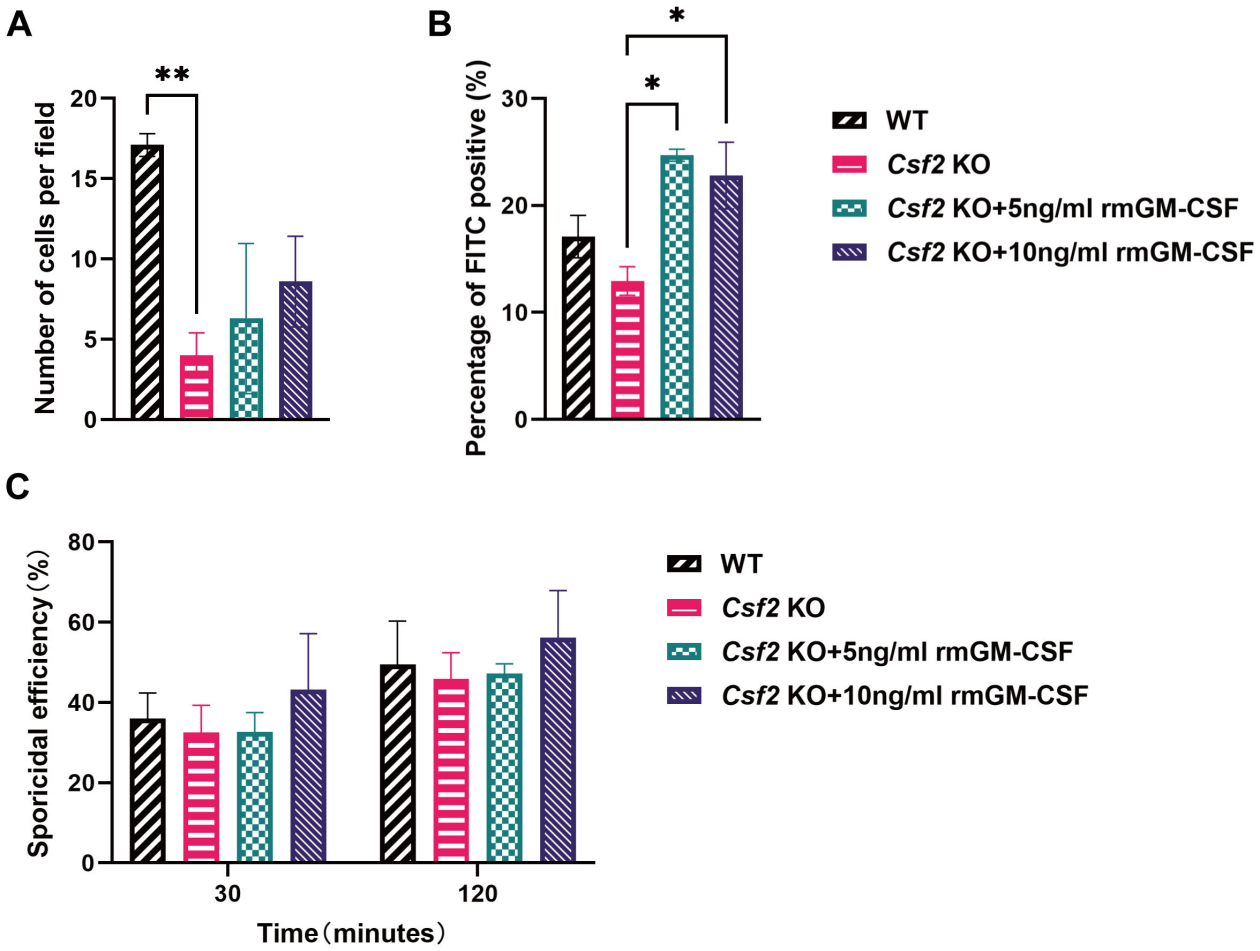
Functional assessment of neutrophils from mice of different genotypes. Neutrophils from the bone marrow of WT and *Csf2* KO mice were assessed for their interaction with *P. verrucosa* conidia. **(A)** Chemotaxis of neutrophils towards conidia (conidia:cells = 10:1) was measured using a transwell chamber after 30 minutes of co-incubation, **P < 0.01. **(B)** FITC positivity in neutrophils (conidia:cells = 1:1) was determined by flow cytometry after 30 minutes of co-incubation, *P < 0.05. **(C)** Killing efficiency of neutrophils against conidia (conidia:cells = 5:1) was evaluated by serial dilution plating following 30 min or 120 min of co-incubation. Data are representative of three independent experiments.

Similar to the assessments conducted with BMDMs, neutrophils were isolated from bone marrow and subjected to corresponding assays. These results indicated that the number of neutrophils migrating towards the conidia was significantly higher in WT mice compared to *Csf2* KO mice, *P* < 0.01 (**Figure 5A**). This finding highlights the critical role of GM-CSF in neutrophil chemotaxis. Furthermore, supplementation with rmGM-CSF only partially restored the chemotactic response of *Csf2* KO neutrophils, however, this restoration did not reach statistical significance.

Additionally, the FITC positivity of neutrophils was evaluated, which reflects their ability to phagocytose conidia. The data showed that after supplementation with rmGM-CSF, the phagocytic activity of *Csf2* KO neutrophils was significantly improved compared to the *Csf2* KO group, both at concentrations of 5 ng/mL and 10 ng/mL, *P* < 0.05 (**Figure 5B**). This indicates that GM-CSF plays a crucial role in enhancing the phagocytic capability of neutrophils.

Finally, the killing efficiency of neutrophils against conidia was assessed(**Figure 5C**). Similar to BMDMs, the absence of GM-CSF did not significantly affect the killing ability of neutrophils against *P. verrucosa* conidia, and GM-CSF supplementation did not enhance neutrophil-mediated conidia killing efficiency.

## 4 Discussion

Dematiaceous fungal infections typically invade through cutaneous wounds or breaches, often progressing to chronic and refractory disease in immunocompromised patients, with potential systemic dissemination. While GM-CSF has been implicated in antifungal defense, its precise mechanistic role remains unclear (6). To address this, the current study established a subcutaneous infection model in WT and *Csf2* KO mice using *P. verrucosa*—a representative dematiaceous fungus causing phaeohyphomycosis—via bilateral hind footpad injection of live conidia to mimic common human infection routes. In both WT and *Csf2* KO mice, footpad tissues exhibited significant swelling and ulceration within 1–2 weeks post-infection. Over time, these symptoms on the footpads gradually resolved, with ulcer scabbing and healing observed in both groups. Remarkably, all infected mice demonstrated a self-limiting disease course, achieving 100% survival by the study endpoint. These findings align with prior observations by Wu et al. (15) in WT mice infected with *P. verrucosa*.

Footpad swelling is commonly used as an indicator of tissue edema and inflammatory infiltration during the inflammatory response. In this study, footpad swelling was evaluated by measuring changes in footpad thickness before and after inoculation in mouse infection models, serving as a metric for assessing the severity of local infection and the intensity of the inflammatory response. The results showed that both WT and *Csf2* KO mice reached peak footpad swelling during the early phase of infection (weeks 1-2), followed by a gradual decline, indicating a progression from mild to severe inflammation and subsequent resolution. However, the swelling rate in *Csf2* KO mice was consistently lower than in WT mice over the 4-week period, suggesting a weaker inflammatory response against *P. verrucosa* in the absence of GM-CSF. To determine whether GM-CSF deficiency affects the host’s ability to clear *P. verrucosa* conidia, the fungal burden in infected footpad tissues was quantified. The findings revealed that *Csf2* KO mice exhibited significantly slower clearance of fungi compared to WT mice, with fungal colonies still detectable at week 4, consistent with histopathological observations. These results indicate that GM-CSF deficiency delays the clearance of pathogenic fungi and prolongs the disease course.

These results on the delayed fungal clearance and altered immune response in *Csf2* KO mice can be interpreted within the broader context of immunity to phaeohyphomycosis. Effective defense requires coordinated innate and adaptive immunity, including C-type lectin receptor (CLR)/CARD9 signaling, neutrophils, macrophages, and Th1/Th17 responses (4, 16–18). This study further demonstrates that GM-CSF deficiency perturbs the cytokine network supporting these immune cells, highlighting its key role in maintaining antifungal immunity against dematiaceous fungi.

During inflammation, monocytes are rapidly mobilized from the bone marrow and recruited to inflamed tissues, where they undergo differentiation into macrophages. Amorim et al. (19) found that type I cytokines GM-CSF and IFN-γ regulate this process. In the study of mouse footpad infections, GM-CSF-deficient mice exhibited higher levels of IFN-γ than WT mice during the first week, likely as a compensatory mechanism for the impaired macrophage differentiation due to GM-CSF deficiency. It is noteworthy that despite local IFN-γ overaccumulation, the serum IFN-γ concentration in *Csf2* KO mice was significantly lower than in WT controls. This may reflect impaired T cell activation in *Csf2* KO mice, consequently affecting sustained IFN-γ production in circulation (20). Additionally, G-CSF levels were observed to be lower in *Csf2* KO mice compared to WT mice. G-CSF, known to promote early granulocyte proliferation and mobilize reserve pools into circulation, is crucial for increasing peripheral blood neutrophil counts and enhancing resistance to fungal infections (21). Consistently, significantly reduced neutrophil infiltration was observed in *Csf2* KO mice. Collectively, these findings suggest that GM-CSF deficiency may reduce neutrophil infiltration through decreased G-CSF production but does not appear to impair macrophage differentiation and recruitment, potentially due to compensatory increases in IFN-γ. These results underscore the pivotal role of GM-CSF in modulating immune responses and its intricate interactions with other cytokines such as IFN-γ and G-CSF.

GM-CSF plays a crucial role in modulating fungal infections by activating monocytes and neutrophils, key components of the innate immune response (22). Macrophages migrate to the infection site via chemotaxis, where they recognize and phagocytize fungal pathogens while producing various pro-inflammatory cytokines and chemokines, including TNFα, CXCL1, CXCL2, CCL2, and IL-1β (23), thereby promoting inflammation and immune defense (24). Neutrophils contribute to anti-fungal immunity not only through phagocytosis and the release of neutrophil extracellular traps (25) but also by secreting granule enzymes, immunomodulatory cytokines, and chemokines, synergizing with other immune components (22). This study further evaluated the impact of GM-CSF on macrophage and neutrophil functions during *P. verrucosa* infection *in vitro*. These results demonstrated that GM-CSF deficiency significantly impaired the chemotactic abilities of both macrophages and neutrophils. Supplementation with 5 ng/mL or 10 ng/mL of rmGM-CSF fully restored the chemotactic defects in macrophages but had no significant effect on neutrophil chemotaxis. However, treatment with rmGM-CSF markedly enhanced the phagocytic capacity of neutrophils against *P. verrucosa* conidia. These findings align with those of Tu et al. (26), who reported that GM-CSF treatment enhances neutrophil phagocytosis in a sepsis model. The data further confirm the similar effects of GM-CSF in combating *P. verrucosa* infection.

Despite uncovering the significant role of GM-CSF in defending against dematiaceous fungal infections and its differential impacts on macrophages and neutrophils, this study has several limitations. First, differences between animal models and the human immune system may limit the direct clinical applicability of these findings. Second, the assessment of immune cell conidial killing activity was conducted within a relatively short timeframe (up to 2 hours) to prioritize the analysis of initial rapid responses and ensure comparability between neutrophils and macrophages. While this approach is well-established, it may not capture potential differences in sustained killing capacity, particularly for macrophages, over longer incubation periods. Third, while this study demonstrates clear functional alterations in GM-CSF–deficient models—such as impaired neutrophil recruitment, defective macrophage chemotaxis, and only partial compensation by elevated IFN-γ—the precise molecular mechanisms driving these phenotypes remain to be elucidated. In particular, it will be important to determine which signaling cascades (e.g., JAK/STAT, PI3K, NF-κB) are disrupted, how GM-CSF shapes transcriptional programs in innate immune cells, and which effector molecules mediate its antifungal activity. Addressing these questions in future studies will be critical for fully clarifying the unique contribution of GM-CSF within the antifungal immune network. Fourth, the WT and *Csf2* KO mice used in this study were not littermates, which is a potential limitation. However, the pure C57BL/6J background of both strains was confirmed, and all mice were co-housed under identical SPF conditions prior to experiments to minimize microbiota variations. Moreover, the concordance between *in vivo* phenotypes and the *in vitro* functional defects observed in KO-derived cells strengthens the conclusion that the observed effects result from GM-CSF deficiency rather than underlying genetic or microbial differences. Additionally, this study focused on the consequences of GM-CSF deficiency and did not directly evaluate whether exogenous GM-CSF could enhance antifungal functions in immunocompetent hosts. This represents a limitation of the present study. Nevertheless, prior research has shown that GM-CSF enhances the antifungal activity of neutrophils and macrophages against major fungal pathogens (12), including *Candida albicans* (27), *Aspergillus fumigatus* (9), and *Cryptococcus neoformans* (28). These precedents provide a strong rationale for future studies to determine whether exogenous GM-CSF can augment immune responses against *P. verrucosa* and serve as a potential adjunctive therapy.

In summary, this study utilized a subcutaneous infection model with *P. verrucosa* in mice to investigate the role and mechanisms of GM-CSF in defending against dematiaceous fungal infections. The current study found that while both WT and *Csf2* KO mice exhibited a tendency towards self-healing, GM-CSF deficiency significantly delayed fungal clearance, prolonged disease duration, and led to reduced local inflammatory responses and neutrophil infiltration. *In vitro* supplementation with rmGM-CSF fully restored macrophage chemotaxis and enhanced neutrophil phagocytosis of *P. verrucosa* conidia, underscoring the critical role of GM-CSF in innate immunity. Additionally, this study revealed complex interactions between GM-CSF and other cytokines, such as IFN-γ and G-CSF, particularly in regulating monocyte/macrophage differentiation and neutrophil mobilization. Despite compensatory increases in IFN-γ levels in *Csf2* KO mice, these were insufficient to overcome the immune defects caused by GM-CSF deficiency, highlighting its irreplaceable role in antifungal immunity. Future research should explore the specific mechanisms of GM-CSF in other dematiaceous fungal infections. It is important to note that the self-limiting nature of this model indicates that GM-CSF’s role is likely preventive or adjunctive in the early phase after inoculation, rather than therapeutic for established disease. Building on this, a key future direction is to explore the synergistic potential of GM-CSF with conventional antifungal drugs, particularly in models of severe, invasive phaeohyphomycosis involving internal organs.

## Data Availability

The raw data supporting the conclusions of this article will be made available by the authors, without undue reservation.

## References

[1] Revankar SG. Dematiaceous fungi. Mycoses. (2007) 50:91–101. doi: 10.1111/j.1439-0507.2006.01331.x, PMID: 17305771

[2] Sánchez-Cárdenas CD Isa-Pimentel M Arenas R. Phaeohyphomycosis: A review. Microbiol Res. (2023) 14:1751–63. doi: 10.3390/microbiolres14040120

[3] He Y Zheng HL Mei H Lv GX Liu WD Li XF. Phaeohyphomycosis in China. Front Cell Infect Microbiol. (2022) 12:895329. doi: 10.3389/fcimb.2022.895329, PMID: 35770068 PMC9235401

[4] Wang X Zhang R Wu W Song Y Wan Z Han W. Impaired specific antifungal immunity in card9-deficient patients with phaeohyphomycosis. J Invest Dermatol. (2018) 138:607–17. doi: 10.1016/j.jid.2017.10.009, PMID: 29080677

[5] Liu X Jiang B Hao H Liu Z. Card9 signaling, inflammation, and diseases. Front Immunol. (2022) 13:880879. doi: 10.3389/fimmu.2022.880879, PMID: 35432375 PMC9005907

[6] Zhang R Wang X Wan Z Li R. CARD9 mutations and related immunological research of one case with disseminated phaeohyphomycosis. J Microbes Infect. (2016) 12:14–23.

[7] Dougan M Dranoff G Dougan SK. Gm-Csf, Il-3, and Il-5 family of cytokines: regulators of inflammation. Immunity. (2019) 50:796–811. doi: 10.1016/j.immuni.2019.03.022, PMID: 30995500 PMC12512237

[8] Barros N Alexander N Viens A Timmer K Atallah N Knooihuizen SAI. Cytokine augmentation reverses transplant recipient neutrophil dysfunction against the human fungal pathogen Candida albicans. J Infect Dis. (2021) 224:894–902. doi: 10.1093/infdis/jiab009, PMID: 33417688 PMC8577195

[9] Kasahara S Jhingran A Dhingra S Salem A Cramer RA Hohl TM. Role of granulocyte-macrophage colony-stimulating factor signaling in regulating neutrophil antifungal activity and the oxidative burst during respiratory fungal challenge. J Infect Dis. (2016) 213:1289–98. doi: 10.1093/infdis/jiw054, PMID: 26908736 PMC4799674

[10] Pruksaphon K Nosanchuk JD Ratanabanangkoon K Youngchim S. Talaromyces marneffei infection: virulence, intracellular lifestyle and host defense mechanisms. J Fungi (Basel Switzerland). (2022) 8:200. doi: 10.3390/jof8020200, PMID: 35205954 PMC8880324

[11] Chen TK Batra JS Michalik DE Casillas J Patel R Ruiz ME. Recombinant human granulocyte-macrophage colony-stimulating factor (Rhu Gm-Csf) as adjuvant therapy for invasive fungal diseases. Open Forum Infect Dis. (2022) 9:ofac535. doi: 10.1093/ofid/ofac535, PMID: 36381625 PMC9645583

[12] Dong Q Wu W Zhang R. Mechanistic insights into granulocyte-macrophage colony-stimulating factor in combating fungal infections: diverse fungal pathogens. Med Mycol. (2025) 63:myaf044. doi: 10.1093/mmy/myaf044, PMID: 40328463

[13] Gao L-J Yu J Wang D-L Li R-Y. Recalcitrant primary subcutaneous phaeohyphomycosis due to *Phialophora verrucosa* . Mycopathologia. (2013) 175:165–70. doi: 10.1007/s11046-012-9602-3, PMID: 23264134

[14] Tong Z Chen SCA Chen L Dong B Li R Hu Z. Generalized subcutaneous phaeohyphomycosis caused by *Phialophora verrucosa*: report of a case and review of literature. Mycopathologia. (2013) 175:301–6. doi: 10.1007/s11046-013-9626-3, PMID: 23392822

[15] Wu W Zhang R Wang X Song Y Liu Z Han W. Impairment of immune response against dematiaceous fungi in Card9 knockout mice. Mycopathologia. (2016) 181:631–42. doi: 10.1007/s11046-016-0029-0, PMID: 27421992

[16] Drummond RA Desai JV Hsu AP Oikonomou V Vinh DC Acklin JA. Human dectin-1 deficiency impairs macrophage-mediated defense against phaeohyphomycosis. J Clin Invest. (2022) 132:e159348. doi: 10.1172/jci159348, PMID: 36377664 PMC9663159

[17] Drummond RA Franco LM Lionakis MS. Human card9: A critical molecule of fungal immune surveillance. Front Immunol. (2018) 9:1836. doi: 10.3389/fimmu.2018.01836, PMID: 30127791 PMC6088205

[18] Wang X Wang W Lin Z Wang X Li T Yu J. Card9 mutations linked to subcutaneous phaeohyphomycosis and Th17 cell deficiencies. J Allergy Clin Immunol. (2014) 133:905–8.e3. doi: 10.1016/j.jaci.2013.09.033, PMID: 24231284

[19] Amorim A De Feo D Friebel E Ingelfinger F Anderfuhren CD Krishnarajah S. Ifnγ and Gm-Csf control complementary differentiation programs in the monocyte-to-phagocyte transition during neuroinflammation. Nat Immunol. (2022) 23:217–28. doi: 10.1038/s41590-021-01117-7, PMID: 35102344

[20] Chen YY Yang WC Chang YK Wang CY Huang WR Li JY. Construction of polycistronic baculovirus surface display vectors to express the Pcv2 Cap(D41) protein and analysis of its immunogenicity in mice and swine. Vet Res. (2020) 51:112. doi: 10.1186/s13567-020-00836-3, PMID: 32907618 PMC7487469

[21] Meng X Zhang H Dong L Min Q Yu M Li Y. Impact of different genetic mutations on granulocyte development and G-Csf responsiveness in congenital neutropenia. Blood Adv. (2024) 8:1667–82. doi: 10.1182/bloodadvances.2023012171, PMID: 38286463 PMC11006815

[22] Damiani G McCormick TS Leal LO Ghannoum MA. Recombinant human granulocyte macrophage-colony stimulating factor expressed in yeast (Sargramostim): A potential ally to combat serious infections. Clin Immunol. (2020) 210:108292. doi: 10.1016/j.clim.2019.108292, PMID: 31676420

[23] Guillot L Carroll SF Homer R Qureshi ST. Enhanced innate immune responsiveness to pulmonary cryptococcus neoformans infection is associated with resistance to progressive infection. Infect Immun. (2008) 76:4745–56. doi: 10.1128/iai.00341-08, PMID: 18678664 PMC2546841

[24] Xu S Shinohara ML. Tissue-resident macrophages in fungal infections. Front Immunol. (2017) 8:1798. doi: 10.3389/fimmu.2017.01798, PMID: 29312319 PMC5732976

[25] Castanheira FVS Kubes P. Neutrophils and nets in modulating acute and chronic inflammation. Blood. (2019) 133:2178–85. doi: 10.1182/blood-2018-11-844530, PMID: 30898862

[26] Tu F Pan L Wu W Cai Y Li J Wang X. Recombinant Gm-Csf enhances the bactericidal ability of Pmns by increasing intracellular Il-1β and improves the prognosis of secondary pseudomonas Aeruginosa pneumonia in sepsis. J Leuk Biol. (2023) 114:443–58. doi: 10.1093/jleuko/qiad088, PMID: 37490847

[27] Hopke A Scherer A Kreuzburg S Abers MS Zerbe CS Dinauer MC. Neutrophil swarming delays the growth of clusters of pathogenic fungi. Nat Commun. (2020) 11:2031. doi: 10.1038/s41467-020-15834-4, PMID: 32341348 PMC7184738

[28] Chen GH Teitz-Tennenbaum S Neal LM Murdock BJ Malachowski AN Dils AJ. Local Gm-Csf-dependent differentiation and activation of pulmonary dendritic cells and macrophages protect against progressive cryptococcal lung infection in mice. J Immunol. (2016) 196:1810–21. doi: 10.4049/jimmunol.1501512, PMID: 26755822 PMC4744503

