# Supplementary material for: The power of GM-CSF: immune regulation in the defense against Phialophora verrucosa infection
Source: Front Immunol. 2025 Oct 20;16:1662183. doi: 10.3389/fimmu.2025.1662183 (PMC12580205; doi:10.3389/fimmu.2025.1662183)
Supplement: Supplementary file 1 [file DataSheet1.pdf]

项目编号：2024NS06-1

上海实验动物研究中心

项目报告

项目名称 C57BL/6J 亚系遗传背景检测

委托单位 上海必凯科翼生物科技有限公司

报告日期 2025 年 06 月 10 日

项目报告

样品名称	C57BL/6J 雄	检验类别	遗传质量检测
型号规格或等级	/		
委托单位名称	上海必凯科翼生物科技有限公司		
检测日期	2025 年 03 月-2025 年 06 月		
样本接收日期	2025 年 3 月 10 日		
受检批数量	1 批		
委托样品数量	数量： C57BL/6J 雄 1 个样本		
检测有效期	/		
检测方案	使用 118 个分布在 20 条染色体上、C57BL/6J 和 C57BL/6NJ 亚系间存在差异的单核苷酸多态性(SNP)位点，基于 PCR 扩增、一代测序获得待测样本各 SNP 位点基因型（检出率≥96%），形成待测小鼠亚系的遗传背景数据。<br>2 个 C57BL/6 亚系的 SNP 参考基因型来源：https://www.informatics.jax.org/snp		
检测结果	使用 118 个 SNP 位点对样本进行检测，检测结果见表 1。来样的 118 个 SNP 位点的基因型都与 C57BL/6J 的参考基因型相同，且都为纯合。		

检测人员：金毅成　报告撰写人：金毅成　报告审核人：赵丽亚　报告批准人：赵芸

日期：2025/06/10　日期：2025/06/10　日期：2025/06/10　日期：2025/06/10

表 1. C57BL/6J 雄在 1~118 号 SNP 位点的检测结果

SNP 序号	染色体号	染色体上的位置 (Mb)	SNP allele	亚系参考基因型		送检样本
				C57BL/6J	C57BL/6NJ	C57BL/6J 雄
1	1	12	T/C	C/C	T/T	C/C
2	1	14	A/T	T/T	A/A	T/T
3	1	19	A/T	A/A	T/T	A/A
4	1	21	C/G	C/C	G/G	C/C
5	1	29	T/C	C/C	T/T	C/C
6	1	58	T/C	T/T	C/C	T/T
7	1	59	A/C	C/C	A/A	C/C
8	1	79	A/G	G/G	A/A	G/G
9	1	95	A/G	G/G	A/A	G/G
10	1	105	C/G	G/G	C/C	G/G
11	1	114	T/C	C/C	T/T	C/C
12	1	135	A/C	C/C	A/A	C/C
13	1	147	T/C	C/C	T/T	C/C
14	1	154	T/C	C/C	T/T	C/C
15	1	164	A/G	G/G	A/A	G/G
16	1	179	A/T	A/A	T/T	A/A
17	2	8	A/T	A/A	T/T	A/A
18	2	11	T/C	C/C	T/T	C/C
19	2	16	A/G	G/G	A/A	G/G
20	2	21	T/G	G/G	T/T	G/G
21	2	23	A/C	A/A	C/C	A/A
22	2	29	A/C	A/A	C/C	A/A
23	2	49	T/C	C/C	T/T	C/C
24	2	77	T/C	C/C	T/T	C/C
25	2	92	T/G	G/G	T/T	G/G
26	2	98	A/T	A/A	T/T	T/T
27	2	108	T/C	T/T	C/C	T/T
28	2	117	A/T	T/T	A/A	T/T
29	2	126	T/C	C/C	T/T	C/C
30	2	146	A/G	A/A	G/G	A/A
31	2	157	T/C	C/C	T/T	C/C
32	2	171	A/G	A/A	G/G	A/A
33	3	5	T/C	C/C	T/T	C/C
34	3	8	A/G	G/G	A/A	G/G
35	3	23	A/T	T/T	A/A(N/A)	T/T
36	3	31	C/G	G/G	C/C	G/G
37	3	55	C/G	G/G	C/C	G/G
38	3	63	T/C	C/C	T/T	C/C
39	3	69	A/G	G/G	A/A	G/G
40	3	141	T/C	C/C	T/T	C/C
41	4	18	T/C	T/T	C/C	T/T

SNP 序号	染色体号	染色体上的位置 (Mb)	SNP allele	亚系参考基因型		样本编号
				C57BL/6J	C57BL/6NJ	C57BL/6J 雄
42	4	27	T/C	T/T	C/C	T/T
43	4	54	T/C	C/C	T/T	C/C
44	4	63	T/C	T/T	C/C	T/T
45	4	100	T/G	T/T	G/G	T/T
46	5	68	A/C	C/C	A/A	C/C
47	5	76	A/G	G/G	A/A	G/G
48	5	86	T/C	C/C	T/T	C/C
49	5	108	T/C	C/C	T/T	C/C
50	5	112	A/G	A/A	G/G	A/A
51	5	143	T/C	T/T	-	T/T
52	5	143	T/G	G/G	T/T	G/G
53	6	27	A/T	T/T	A/A	T/T
54	6	38	A/G	G/G	A/A	G/G
55	6	58	A/G	A/A	G/G	A/A
56	6	64	A/T	T/T	A/A	T/T
57	6	71	A/C/T	C/C	A/A	C/C
58	6	108	A/T/G	G/G	A/A	G/G
59	7	98	T/C	C/C	T/T	C/C
60	7	123	A/G	A/A	G/G	A/A
61	7	127	A/G	A/A	G/G	A/A
62	7	134	A/T	A/A	T/T	A/A
63	8	15	T/C	T/T	C/C	T/T
64	8	22	A/G	G/G	A/A	G/G
65	8	56	A/G	G/G	A/A	G/G
66	8	76	A/G	G/G	A/A	G/G
67	8	90	A/G	G/G	A/A	G/G
68	8	114	A/G	G/G	A/A	G/G
69	9	20	T/C	T/T	C/C	T/T
70	9	25	A/G	A/A	G/G	A/A
71	9	58	A/G	G/G	A/A	G/G
72	9	111	T/C	C/C	T/T	C/C
73	10	10	A/T/G	G/G	T/T	G/G
74	10	32	T/C	T/T	C/C	T/T
75	10	40	A/G	G/G	A/A	G/G
76	10	47	T/G	G/G	T/T	G/G
77	10	55	T/C	T/T	C/C	T/T
78	10	56	A/G	G/G	A/A	G/G
79	10	64	T/C	T/T	C/C	T/T
80	10	77	A/T/C	A/A	T/T	A/A
81	10	84	T/C	T/T	C/C	T/T
82	11	4	A/G	G/G	A/A	G/G
83	11	12	A/T	A/A	T/T	A/A
84	11	44	A/G	G/G	A/A	G/G

序号	染色体号	染色体上的位置 (Mb)	SNP allele	亚系参考基因型		样本编号
				C57BL/6J	C57BL/6NJ	C57BL/6J 雄
85	11	46	T/C/G	T/T	C/C	T/T
86	11	75	T/G	G/G	T/T	G/G
87	11	86	T/C	C/C	T/T	C/C
88	12	29	T/G	T/T	G/G	T/T
89	12	38	A/G	G/G	-	G/G
90	12	47	A/G	A/A	G/G	A/A
91	12	54	A/T	T/T	T/T	T/T
92	12	82	A/G	G/G	A/A	G/G
93	12	93	A/T	A/A	T/T	A/A
94	13	6	A/G	A/A	G/G	A/A
95	13	26	A/G	A/A	G/G	A/A
96	13	40	T/C	T/T	C/C	T/T
97	13	62	T/C	C/C	T/T	C/C
98	14	68	T/C	G/G	T/T	G/G
99	14	72	A/G	G/G	A/A	G/G
100	14	113	A/G	G/G	A/A	G/G
101	14	119	T/C	C/C	T/T	C/C
102	15	22	T/G	T/T	G/G	T/T
103	15	53	T/C	C/C	T/T	C/C
104	15	54	A/G	A/A	G/G	A/A
105	16	16	T/C	T/T	C/C	T/T
106	16	19	T/C	C/C	T/T	C/C
107	16	58	A/G	G/G	A/A	G/G
108	17	58	T/C	T/T	C/C	T/T
109	17	66	T/C	C/C	T/T	C/C
110	18	19	C/G	G/G	C/C	G/G
111	18	22	T/G	G/G	T/T	G/G
112	18	40	T/C	T/T	C/C	T/T
113	18	86	A/G	A/A	G/G	A/A
114	19	22	T/C	C/C	T/T	C/C
115	19	38	A/G	G/G	A/A	G/G
116	19	48	T/G	G/G	T/T	G/G
117	X	106	A/G	A/A	G/G	A/A
118	X	138	A/T	A/A	T/T	A/A

"-"表示该位点在网上没有查到数据

以上为本次检测报告的全部内容
