# Supplementary material for: The power of GM-CSF: immune regulation in the defense against Phialophora verrucosa infection
Source: Front Immunol. 2025 Oct 20;16:1662183. doi: 10.3389/fimmu.2025.1662183 (PMC12580205; doi:10.3389/fimmu.2025.1662183)
Supplement: Supplementary file 2 [file DataSheet2.docx]

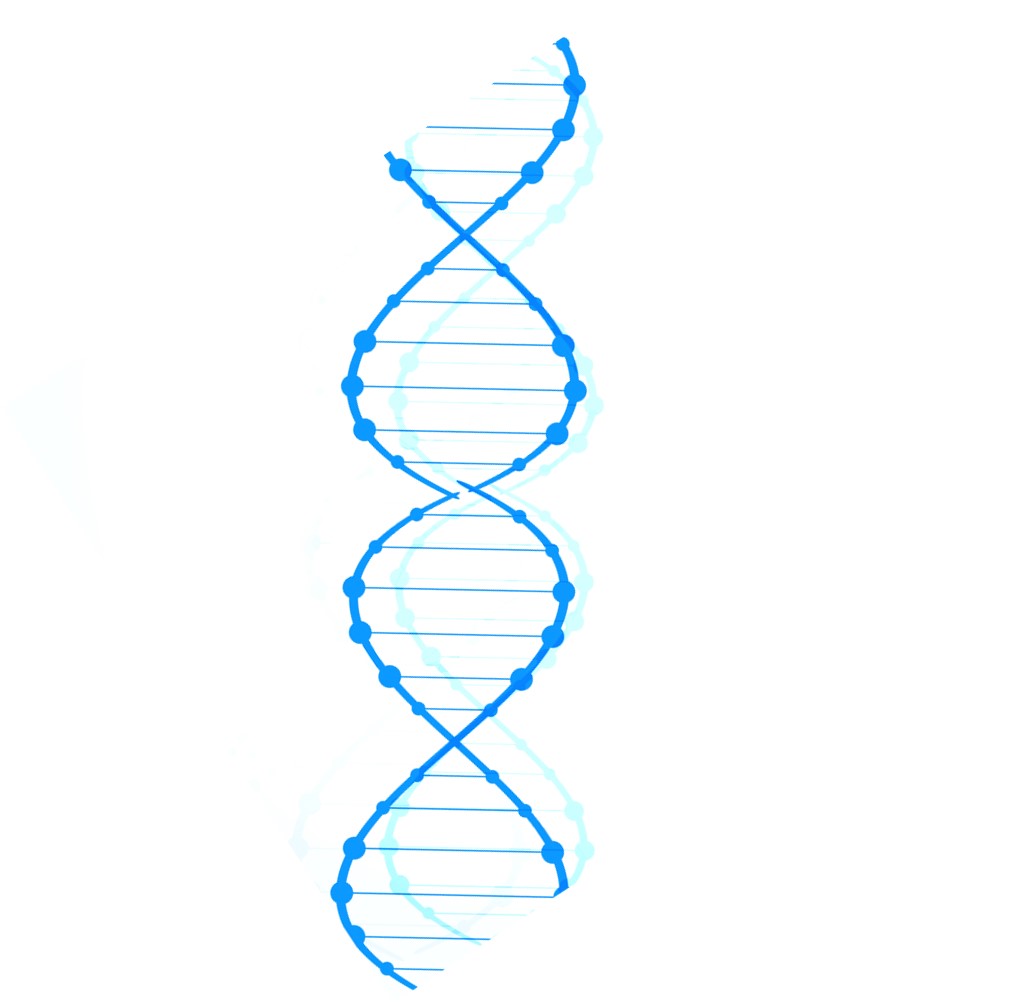

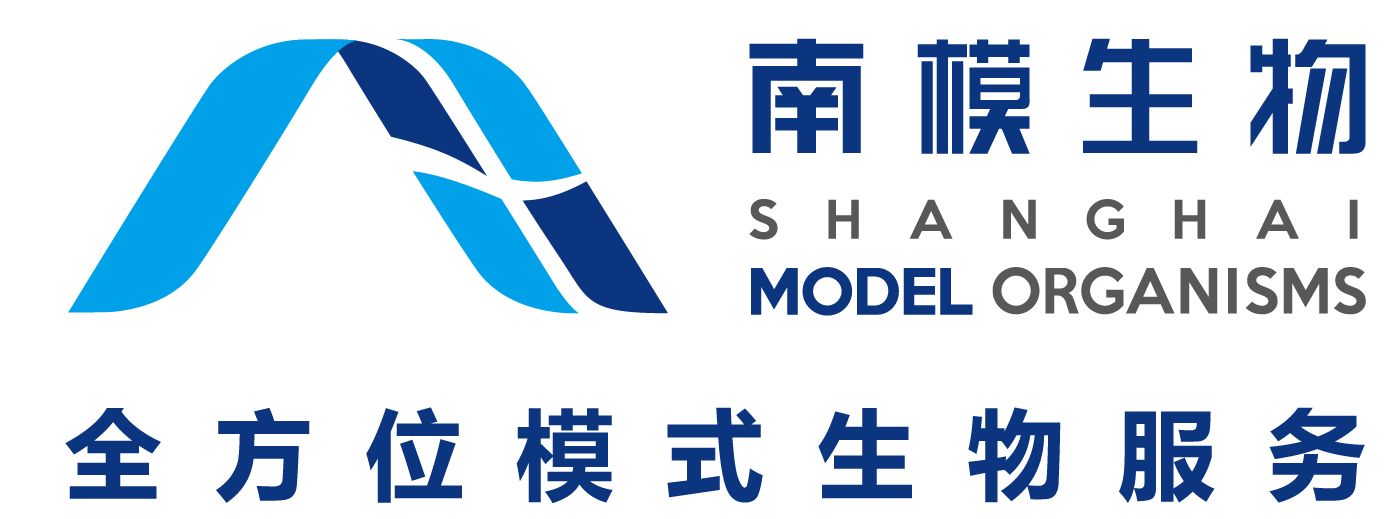


**Csf2-eKO1基因敲除小鼠模型结题报告**

**目录**

[1. 项目内容 2](#_Toc25138428)

[1.1 项目目标 2](#_Toc25138429)

[1.2 项目基本信息 2](#_Toc25138430)

[2. 摘要 2](#_Toc25138431)

[3. 设计策略 3](#_Toc25138432)

[3.1设计策略示意图： 3](#_Toc25138433)

[3.2 gRNAs序列信息 3](#_Toc25138434)

[4. 结果 4](#_Toc25138435)

[4.1 F0代小鼠基因型鉴定 4](#_Toc25138436)

[4.1.1 F0代小鼠基因型鉴定策略 4](#_Toc25138437)

[4.1.2 阳性F0代小鼠PCR测序鉴定结果 5](#_Toc25138438)

[4.1.3阳性F0代小鼠PCR鉴定方法 6](#_Toc25138439)

[4.2 F1代小鼠获得及基因型鉴定 7](#_Toc25138440)

[4.2.1 基因敲除F1代小鼠基因型比较分析 7](#_Toc25138441)

[5. 小鼠交付后，后续相关工作流程 11](#_Toc25138442)

[5.1 小鼠验证 11](#_Toc25138443)

[5.2 后续小鼠繁殖方案 11](#_Toc25138444)

[5.2.1 后续实验小鼠繁殖简要流程及建议： 12](#_Toc25138445)

[5.3 后续小鼠基因型鉴定方案 12](#_Toc25138446)

[5.3.1 敲除小鼠基因型鉴定方案（用于鉴定纯合子、杂合子和野生型） 14](#_Toc25138447)

1. 项目内容

## 1.1 项目目标

利用CRISPR/Cas9技术，获得Csf2基因敲除的杂合子小鼠。

**1.2 项目基本信息**

目的基因名称（Ensembl号）：Csf2（ENSMUSG00000018916）

目的基因Ensembl网址链接：<http://www.ensembl.org/Mus_musculus/geneview?gene=ENSMUSG00000018916>

方案针对的转录本（Ensembl号）：Csf2-201（ENSMUST00000019060.5）

Flox针对的exon：exon 1-3

2. 摘要

本项目采用CRISPR/Cas9技术，利用非同源重组修复引入突变的方式，造成Lyve1基因蛋白读码框移码，功能缺失。简要过程如下：通过体外转录的方式，获得Cas9 mRNA和gRNA；将Cas9 mRNA和gRNA显微注射到C57BL/6J小鼠的受精卵中，获得F0代小鼠。PCR扩增及测序鉴定阳性的F0代小鼠与C57BL/6J小鼠交配获得5只阳性F1代小鼠。

3. 设计策略

## 3.1设计策略示意图：


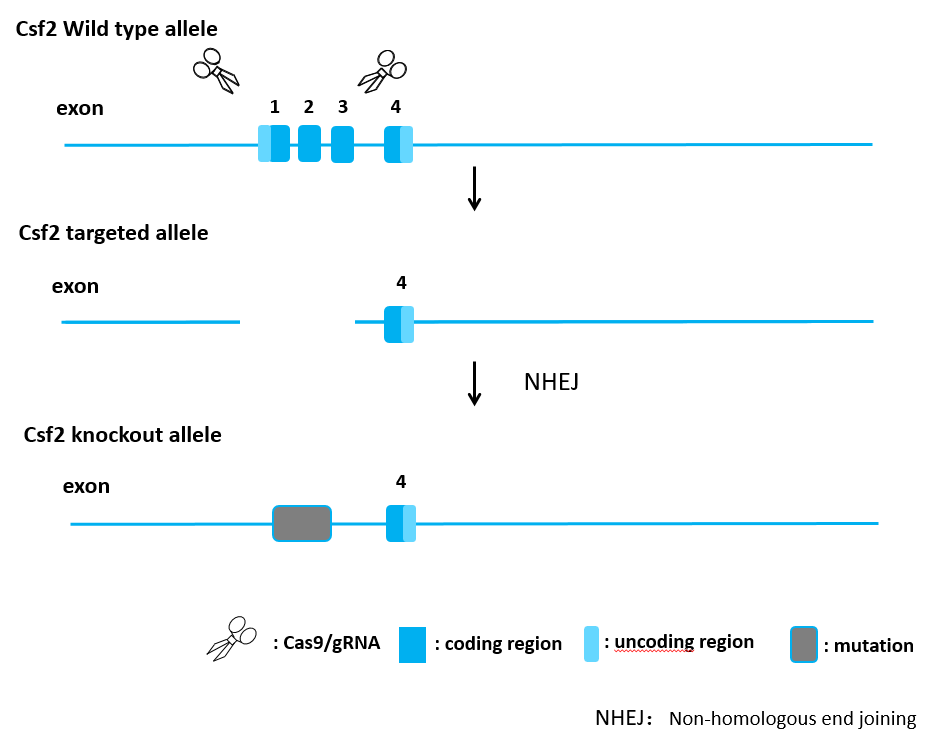


**图1**：小鼠构建策略示意图

## 3.2 gRNAs序列信息

| gRNAs | Sequence (5’-3’) |
| --- | --- |
| gRNA1 | AATGGTGACCACAGAACTCC AGG |
| gRNA2 | ATGGTGACCACAGAACTCCA GGG |
| gRNA3 | CCCTCCCAGACTAAGACCTG GGG |
| gRNA4 | GTACACAGCATCAGGGCCCC AGG |

# **4.** 结果

## 4.1 F0代小鼠基因型鉴定

将注射后的受精卵移植到假孕母鼠中，20天左右出生的小鼠为F0代小鼠。通过PCR扩增及测序对其进行基因型鉴定。由于受精卵早期卵裂速度很快，因此得到的F0 代小鼠为嵌合体，不一定具备稳定遗传的能力，需要进行传代以获得可稳定遗传的F1 代小鼠。

### 4.1.1 F0代小鼠基因型鉴定策略

F0代小鼠基因型鉴定策略如下图所示：


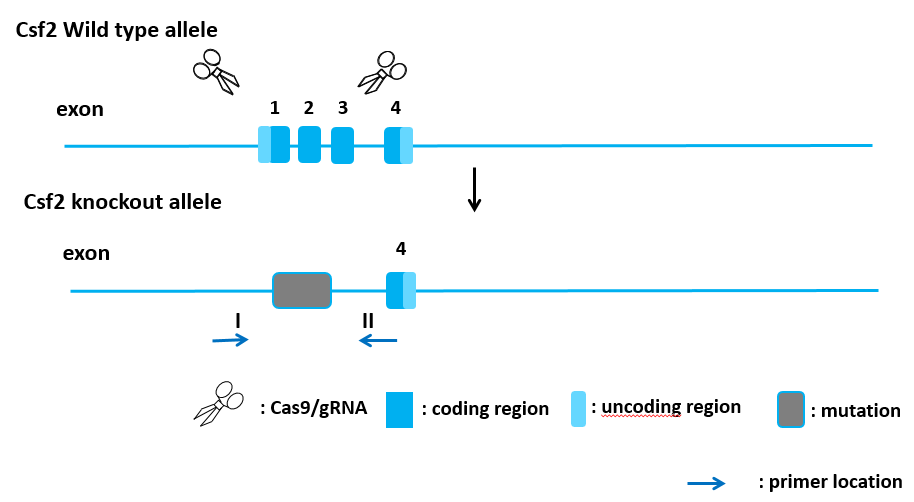


**图2**：F0代小鼠鉴定策略示意图

### 4.1.2 阳性F0代小鼠PCR测序鉴定结果

F0代小鼠PCR产物，经测序，阳性F0代阳性小鼠为：3、4、6号，突变后的基因组序列如表2所示：

表2：F0代小鼠序列突变情况。

| 鼠号 | 序列信息（5’—3’） | 突变情况 |
| --- | --- | --- |
| WT | agattccacaactcaggtagttcccccgcccccctggagttctgtggtcaccattaatcatttcctc……gtgagtacacagcatcagggccccaggtcttagtctgggagggtgcggccagtaaggagagacttctggctgtagc | Wild type |
| 3、4、6 | AGATTCCACAACTCAGGTAGTTCCCCCGCCCCCCTGG…(-1312bp)…GAGGGTGCGGCCAGTAAGGAGAGACTTCTGGCTGTAGC | -1312bp |
| 3、4、6 | AGATTCCACAACTCAGGTAGTTCCCCCGCCCCCCTGGG…G(-1305bp)…AGTCTGGGAGGGTGCGGCCAGTAAGGAGAGACTTCTGGCTGTAGC | -1305+1bp |

### 4.1.3阳性F0代小鼠PCR鉴定方法

引物信息：

|  | **Sequence 5' --> 3'** | **Primer Type** |
| --- | --- | --- |
| I | AGGTGGCTGGAAAGAGAACG | Forward |
| II | TGACATGGGCAGATGGTTCC | Reverse |

反应体系：

| \| Reaction Component \| Volume (µl) \| \| --- \| --- \| \| ddH2O \| 13.2 \| \| GXL PCR Buffer \| 2 \| \| 2.5 mM dNTP \| 2 \| \| Primer I (10pmol/µl) \| 0.5 \| \| Primer II (10pmol/µl) \| 0.5 \| \| GXL DNA Polymerase* \| 0.8 \| \| Tail genomic DNA \| 1 \| \| Total \| 20 \| |
| --- | --- | --- | --- | --- | --- | --- | --- | --- | --- | --- | --- | --- | --- | --- | --- | --- | --- | --- |

* PrimeStar GXL（TaKaRa，Code No：R050A）

反应条件：

| Step # | | | Temp (℃) | Time | Note |
| --- | --- | --- | --- | --- | --- |
| 1 | | | 94 | 3 min | - |
| 2 | | | 98 | 15 sec | - |
| 3 | | | 60 | 15 sec | - |
| 4 | | | 68 | 4 min | repeat steps 2-4 for 35cycles |
| 5 | | | 68 | 5 min | - |
| 6 | | | 12 | - | hold |
|  |  |  |  |  |  |

## 4.2 F1代小鼠获得及基因型鉴定

阳性的**F0**代小鼠与野生型C57BL/6J小鼠交配，获得的F1代杂合子小鼠有2种类型，分别为：敲除类型1，缺失1312个碱基对；敲除类型2，缺失1305个碱基对并插入1个碱基对。鉴定方法及同4.1F0代小鼠鉴定。最终提供的F1代杂合子小鼠信息如下：

敲除类型1：

| 鼠号 | 出生日期 | 代数 | 性别 | 类型 | 基因型 | 突变类型 | 亲代 | |
| --- | --- | --- | --- | --- | --- | --- | --- | --- |
| Mice ID | DOB | Gener-ations | Sex | Type | Geno-type | mutation type | Father | Mother |
| 3 | 2020/11/24 | F1 | ♂ | KO | He | -1312 | 3# 4# 6# | WT |
| 9 | 2020/11/24 | F1 | ♂ | KO | He | -1312 | 3# 4# 6# | WT |
| 26 | 2020/11/24 | F1 | ♀ | KO | He | -1312 | 3# 4# 6# | WT |
| 29 | 2020/11/24 | F1 | ♀ | KO | He | -1312 | 3# 4# 6# | WT |

敲除类型2：

| 鼠号 | 出生日期 | 代数 | 性别 | 类型 | 基因型 | 突变类型 | 亲代 | |
| --- | --- | --- | --- | --- | --- | --- | --- | --- |
| Mice ID | DOB | Gener-ations | Sex | Type | Geno-type | mutation type | Father | Mother |
| 14 | 2020/11/24 | F1 | ♀ | KO | He | -1305+1 | 3# 4# 6# | WT |
| 15 | 2020/11/24 | F1 | ♀ | KO | He | -1305+1 | 3# 4# 6# | WT |
| 23 | 2020/11/24 | F1 | ♂ | KO | He | -1305+1 | 3# 4# 6# | WT |

### 4.2.1 基因敲除F1代小鼠基因型比较分析

**4.2.1.1 敲除类型1基因型比较分析**

**4.2.1.1.1 敲除前后序列比对：**

WT:

agattccacaactcaggtagttcccccgcccccctggagttctgtggtcaccattaatcatttcctc……gtgagtacacagcatcagggccccaggtcttagtctgggagggtgcggccagtaaggagagacttctggctgtagc

MT:

AGATTCCACAACTCAGGTAGTTCCCCCGCCCCCCTGG…(-1312bp)…GAGGGTGCGGCCAGTAAGGAGAGACTTCTGGCTGTAGC

**4.2.1.1.2敲除前后测序结果比对：**


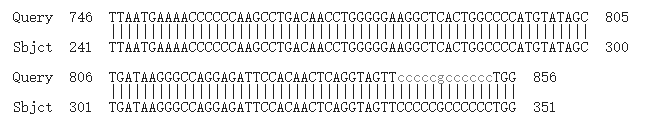


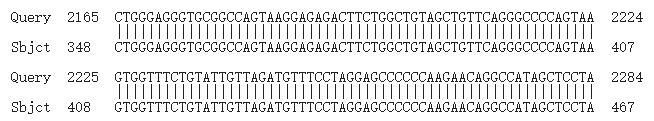


Query为野生型基因组序列，Subject为实际测序结果。

**4.2.1.1.3 敲除前后基因功能分析：**

在该品系小鼠中，基因敲除后，目的基因缺失，从而造成基因功能缺失。

**4.2.1.2 敲除类型2基因型比较分析**

**4.2.1.2.1 敲除前后序列比对：**

WT:

agattccacaactcaggtagttcccccgcccccctggagttctgtggtcaccattaatcatttcctc……gtgagtacacagcatcagggccccaggtcttagtctgggagggtgcggccagtaaggagagacttctggctgtagc

MT:

AGATTCCACAACTCAGGTAGTTCCCCCGCCCCCCTGGG…G(-1305bp)…AGTCTGGGAGGGTGCGGCCAGTAAGGAGAGACTTCTGGCTGTAGC

**4.2.1.2.2敲除前后测序结果比对：**


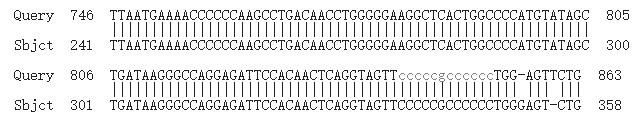


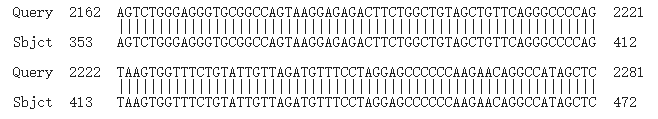


Subject为野生型基因组序列，Query为实际测序结果。

**4.2.1.2.3 敲除前后基因功能分析：**

在该品系小鼠中，基因敲除后，目的基因缺失，从而造成基因功能缺失。

5. 小鼠交付后，后续相关工作流程

## 5.1 小鼠验证

客户接收到我司交付的小鼠后，请首先按照送货单上交付小鼠的信息，对交付小鼠数量和交付小鼠的编号进行核对。我司小鼠的编号原则如下图所示：


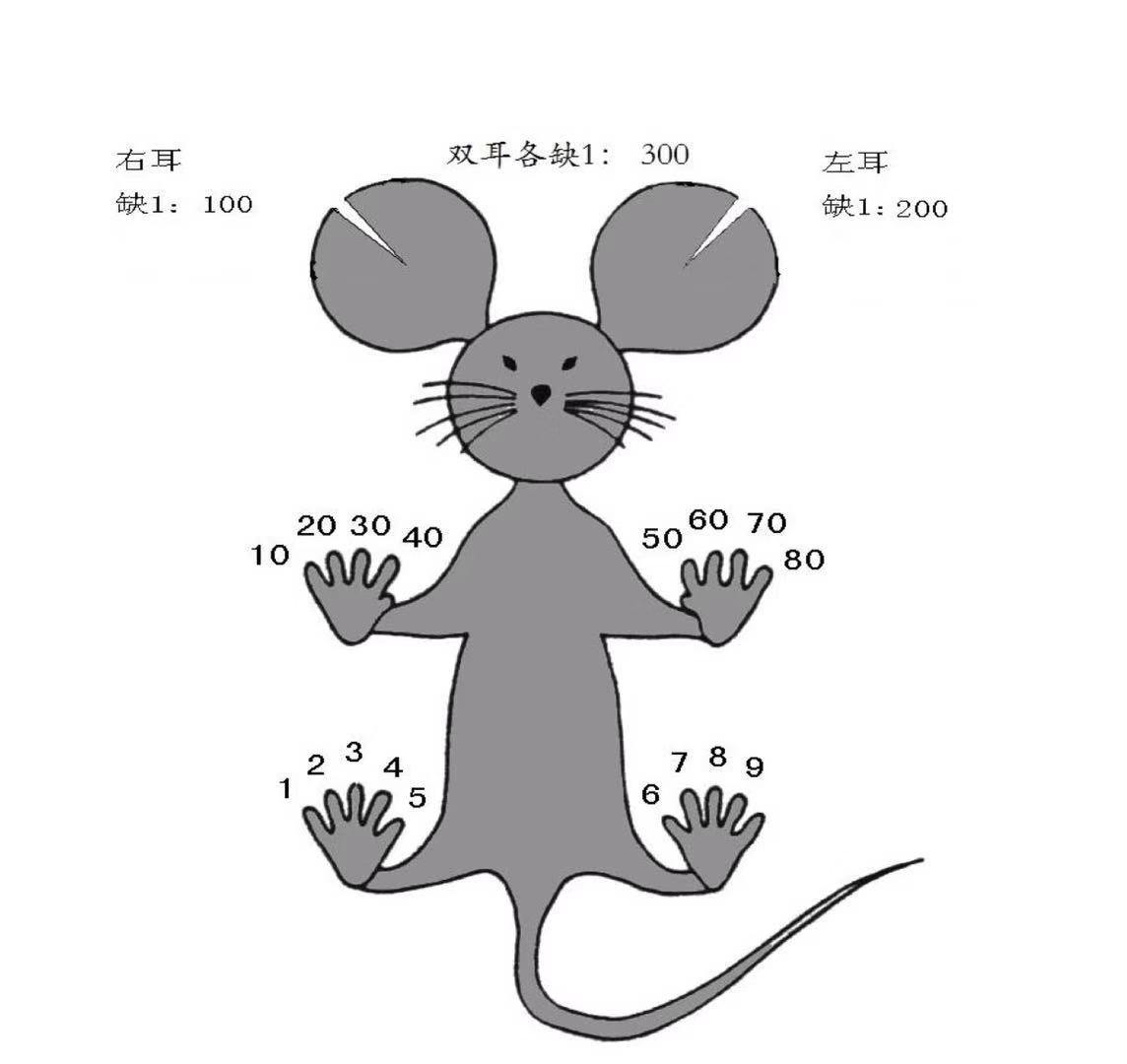


## 5.2 后续小鼠繁殖方案

我司交付的小鼠，如无特殊说明，均为基因敲除杂合子小鼠（gene^+/-^）。

### 5.2.1 后续实验小鼠繁殖简要流程及建议：

**5.2.1.1 后续实验小鼠繁殖简要流程：**

将获得的基因敲除杂合子小鼠（gene^+/-^）分成两部分：一部分杂合子小鼠与野生型小鼠交配，扩群繁育较多的杂合子小鼠；一部分杂合子小鼠自交，获得基因敲除纯合子小鼠（gene^-/-^），进行基因敲除效果验证和后续的表型分析。

注：上述实验小鼠的繁育方案仅为一个参考方案，小鼠繁殖过程中，因各种基因型小鼠的数量，情况各不相同，请根据实际情况情况进行自行调整。

## 5.3 后续小鼠基因型鉴定方案

交付的小鼠经验证无问题后，在后续小鼠交配繁殖过程中，其鉴定方案，根据突变小鼠的碱基变化情况不同，有以下几种供参考。

**方案一：PCR产物测序**

适用范围：几乎适用于所有不能直接通过PCR产物大小和有无判断基因型的情况，尤其适用于碱基突变<5bp的情况；

优点：直接，可靠性高；

缺点：费用高（增加了测序成本），但较方案二和方案三，成本不一定高。

**方案二：PCR产物有无**

适用范围：通常适用于碱基突变>5bp的情况，将其中一条引物设计在发生突变的区域，利用针对野生型引物只能扩增出野生型条带，针对突变型引物只能扩增出突变型条带的原理，区分野生型、杂合子和纯合子基因型；

优点：成本低；

缺点：引物设计位点受限制较大，可能有假阳性情况。

**方案三：PCR产物大小**

适用范围：通常适用于碱基突变>40bp的情况，将鉴定引物设计在发生突变的区域两侧，利用野生型和突变型PCR产物大小的差异，区分野生型、杂合子和纯合子基因型；

优点：成本低，直接；

缺点：PCR产物大小不易过大，引物设计可能受限。

**方案四：PCR产物限制性内切酶酶切**

适用范围：适用于突变产生了新的酶切位点，或导致原有的酶切结果条带大小发生明显变化；

优点：可能成本较方案一低一些；

缺点：步骤繁琐，有假阳性、假阴性情况。

**方案五：PCR产物T7核酸内切酶I酶切**

适用范围：适用于所有突变类型；

优点：适用范围广；

缺点：无法区分野生型和纯合子，费用高。

**方案六：高分辨率溶解曲线分析（High-resolution melting, HRM）**

适用范围：几乎适用于所有情况；

优点：适用范围广；

缺点：依赖于仪器，费用高，间接结果。

本项目所获得的基因敲除小鼠品系，后续小鼠繁育过程中，小鼠基因型鉴定所需引物及条件，本报告给出了其中的一套方案。如上所述，小鼠基因型鉴定可以有多种方案，客户也可根据我们给出的设计原则，对鉴定方案进行自行设计。引物设计软件无特殊要求，常规的DNASTAR软件中的PrimerSelect程序等均可满足要求（相关软件可通过网络搜索下载）。

### 5.3.1 敲除小鼠基因型鉴定方案（用于鉴定纯合子、杂合子和野生型）

敲除小鼠基因型鉴定方案为：PCR产物大小。(以类型1为例)


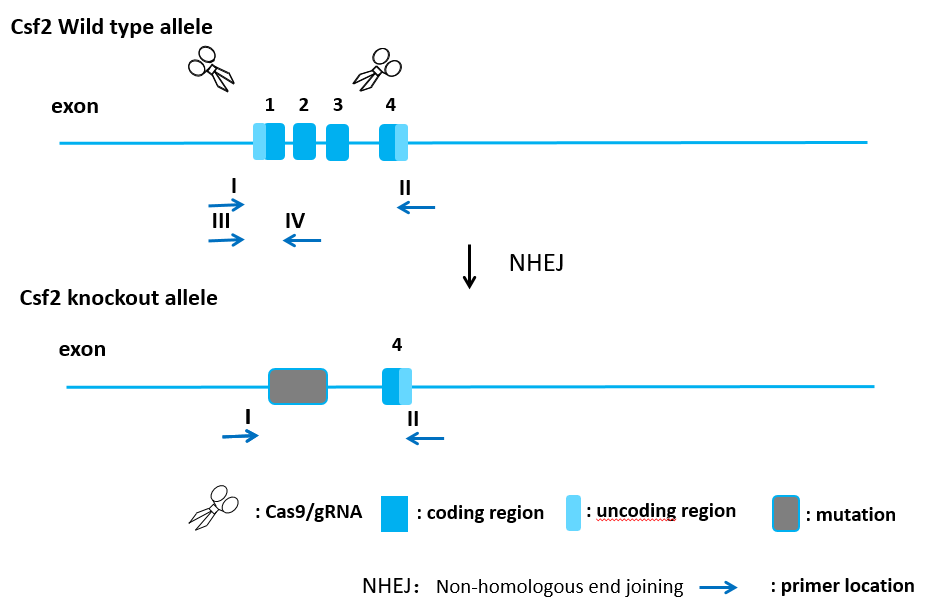


**5.3.1.1 PCR鉴定条件**

| **Primer** | **Sequence (5’→3’)** | | | | **Primer type** |
| --- | --- | --- | --- | --- | --- |
| **P1** | AGGTGGCTGGAAAGAGAACG | | | | Forward |
| **P2** | TGACATGGGCAGATGGTTCC | | | | Reverse |
| **P3** | AGGTGGCTGGAAAGAGAACG | | | | Forward |
| **P4** | GGGCTTCTTTGATGGCCTCT | | | | Reverse |
| **PCR Reaction System** | **Reaction Component** | | | | **Volume (µl)** |
|  | ddH2O | | | | 14.9 |
|  | 10 x Taq PCR Buffer | | | | 2 |
|  | 2.5 mM dNTP | | | | 1 |
|  | Primer I (10pmol/µl) | | | | 0.5 |
|  | Primer II (10pmol/µl) | | | | 0.5 |
|  | Taq DNA Polymerase* | | | | 0.1 |
|  | genomic DNA | | | | 1 |
|  | Total | | | | 20 |
|  |  | | | |  |
|  | ddH2O | | | | 14.9 |
|  | 10 x Taq PCR Buffer | | | | 2 |
|  | 2.5 mM dNTP | | | | 1 |
|  | Primer III (10pmol/µl) | | | | 0.5 |
|  | Primer IV (10pmol/µl) | | | | 0.5 |
|  | Taq DNA Polymerase* | | | | 0.1 |
|  | genomic DNA | | | | 1 |
|  | Total | | | | 20 |
|  | *Taq DNA Polymerase from Takara (Code number: R001A) | | | | |
| **Cycling Reaction** | **Step** | **Temp（℃）** | **Time** | **Note** | |
|  | 1 | 95 | 5 min |  | |
|  | 2 | 95 | 15 sec |  |  |
|  | 3 | 60 | 15 sec |  |  |
|  | 4 | 72 | 4min | repeat steps 2-4 for 35cycles | |
|  | 5 | 72 | 5 min |  | |
|  | 6 | 12 | Hold |  |  |
| **Result** | PCR Products:  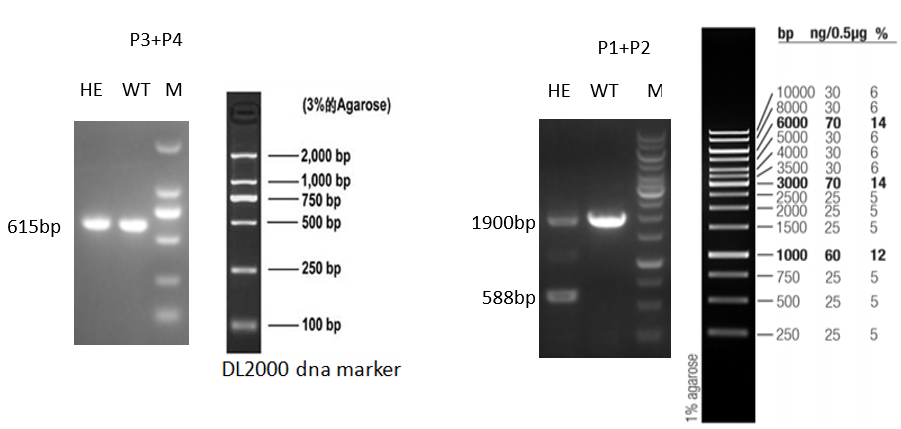  Separated by gel electrophoresis on a 1 % agarose gel. | | | | |
| **Genotype** | 野生型：P1和P2 PCR获得单一的1900bp片段；P3和P4可以获得615bp片段  杂合子：P1和P2 PCR获得588bp和1900bp两个片段；P3和P4可以获得615bp的片段。  纯合子：P1和P2 PCR获得单一的588bp片 段；P3和P4不能获得条带。 | | | | |

Note: 在野生型及杂合子小鼠中，P1、P2引物是否能扩增出大条带并不影响结果判读，因为设计这对引物目的是用于扩增KO条带。
