# Supplementary material for: The power of GM-CSF: immune regulation in the defense against Phialophora verrucosa infection
Source: Front Immunol. 2025 Oct 20;16:1662183. doi: 10.3389/fimmu.2025.1662183 (PMC12580205; doi:10.3389/fimmu.2025.1662183)
Supplement: Supplementary file 3 [file DataSheet3.pdf]

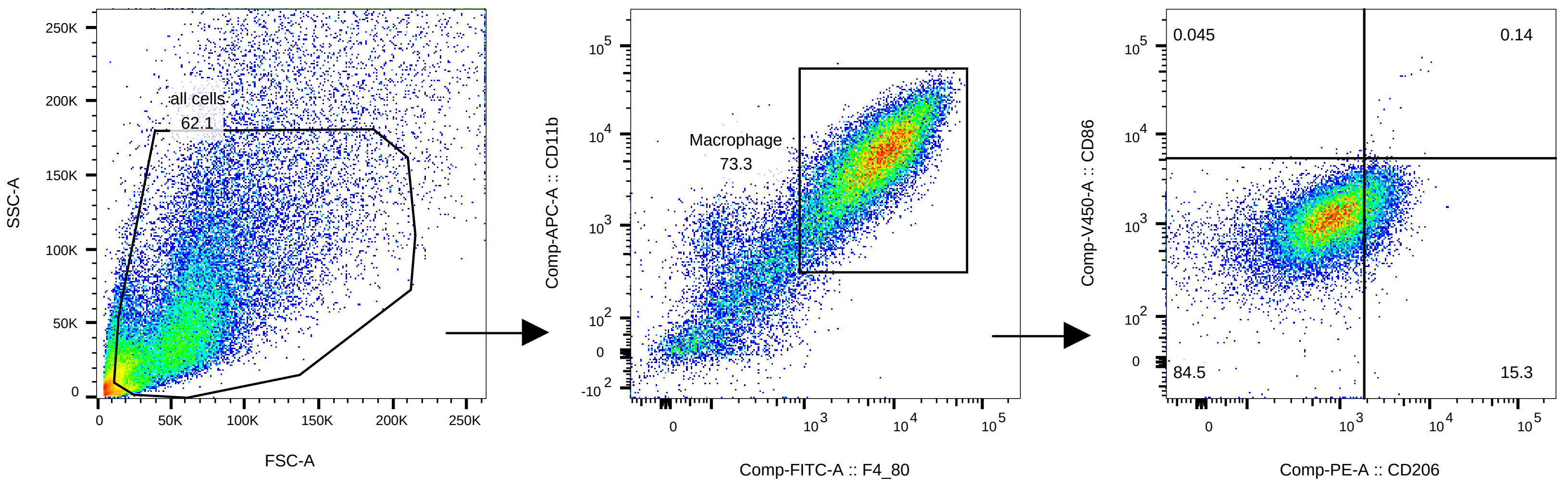

Specimen_001_CSf2-1_012.fcs
Ungated
48318

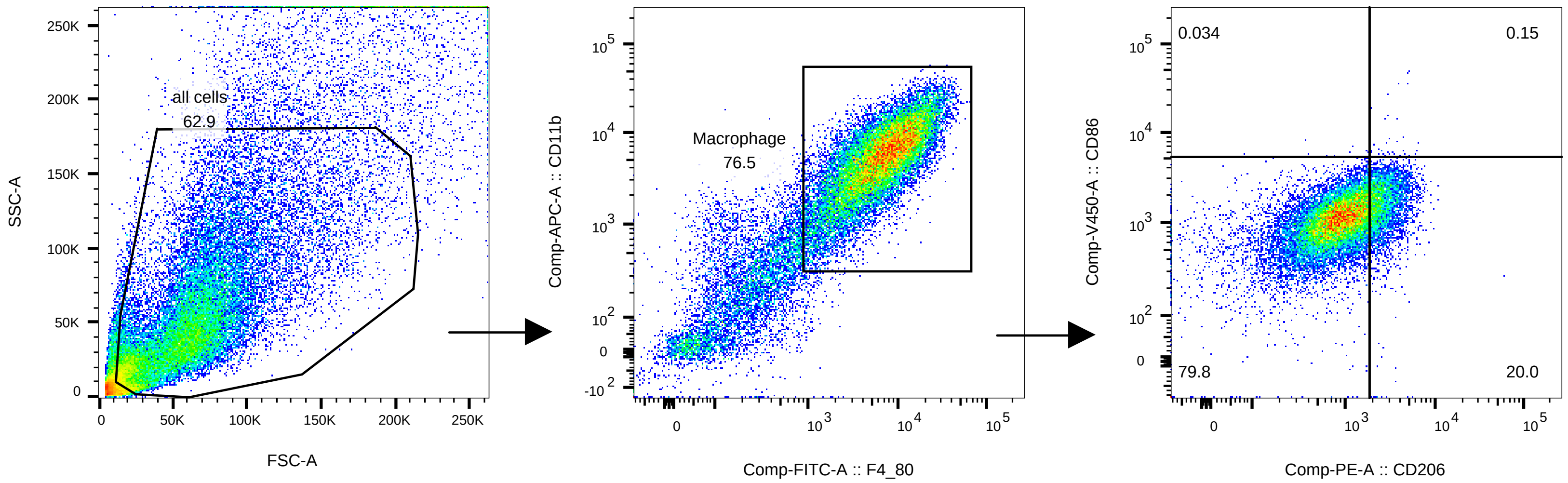

Specimen_001_CSf2-2_013.fcs
Ungated
48657

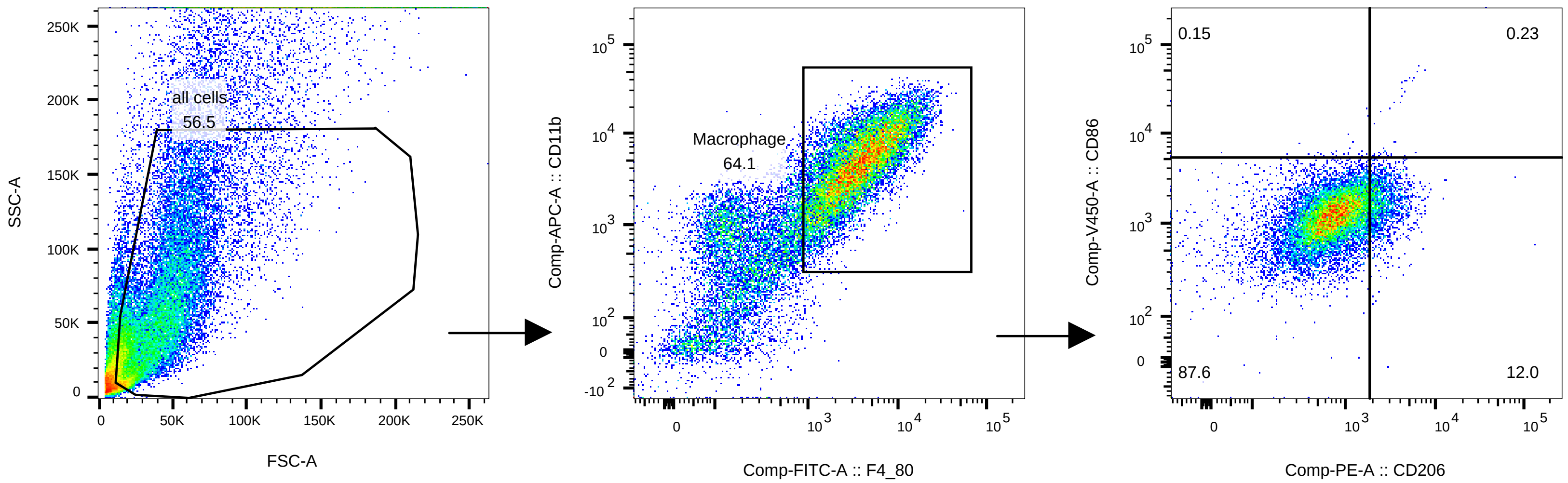

Specimen_001_WT-1_003.fcs
Ungated
42820

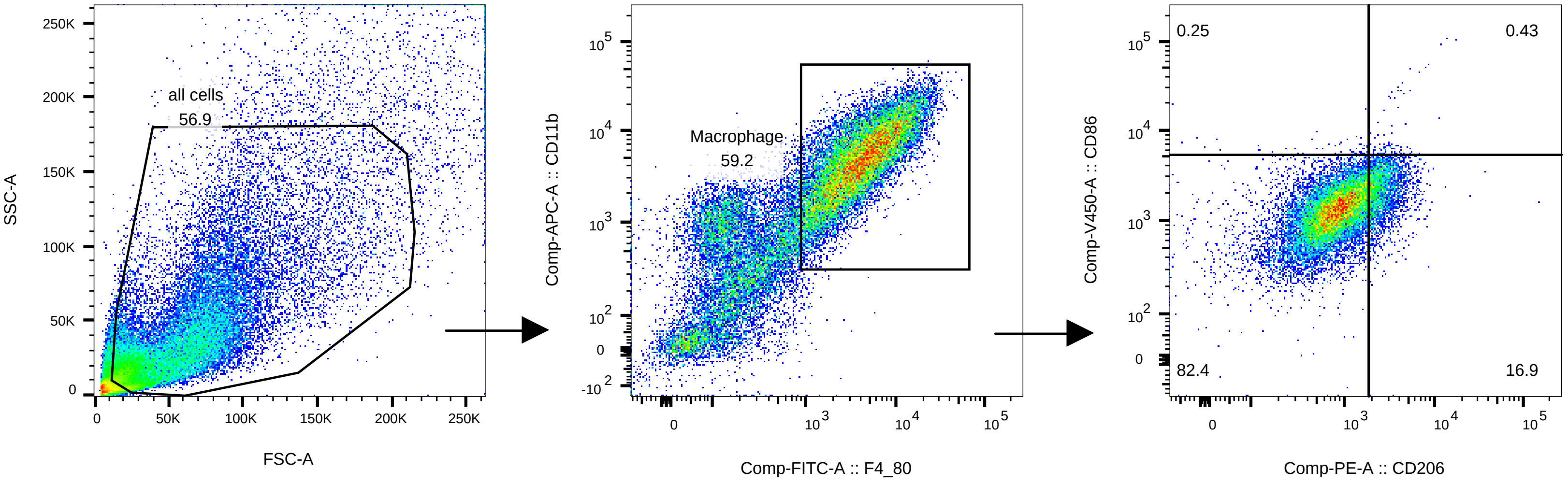

Specimen_001_WT-2_004.fcs
Ungated
55871
