# Supplementary material for: The power of GM-CSF: immune regulation in the defense against Phialophora verrucosa infection
Source: Front Immunol. 2025 Oct 20;16:1662183. doi: 10.3389/fimmu.2025.1662183 (PMC12580205; doi:10.3389/fimmu.2025.1662183)
Supplement: Supplementary file 4 [file DataSheet4.pdf]

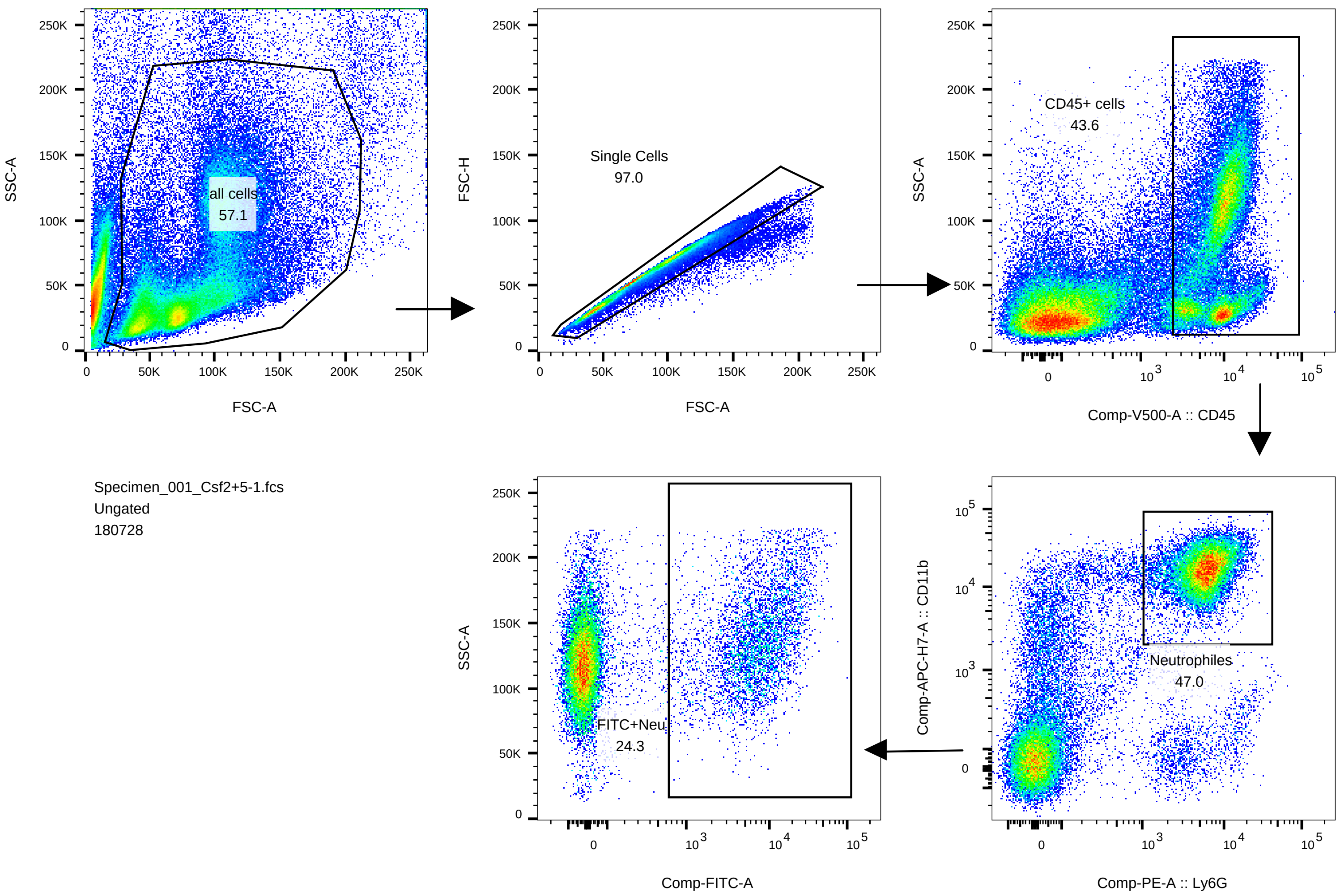
all cells
57.1
SSC-A
FSC-A
Single Cells
97.0
FSC-H
FSC-A
CD45+ cells
43.6
SSC-A
Comp-V500-A :: CD45
Specimen_001_Csf2+5-1.fcs
Ungated
180728
FITC+Neu
24.3
SSC-A
Comp-FITC-A
Neutrophiles
47.0
Comp-APC-H7-A :: CD11b
Comp-PE-A :: Ly6G

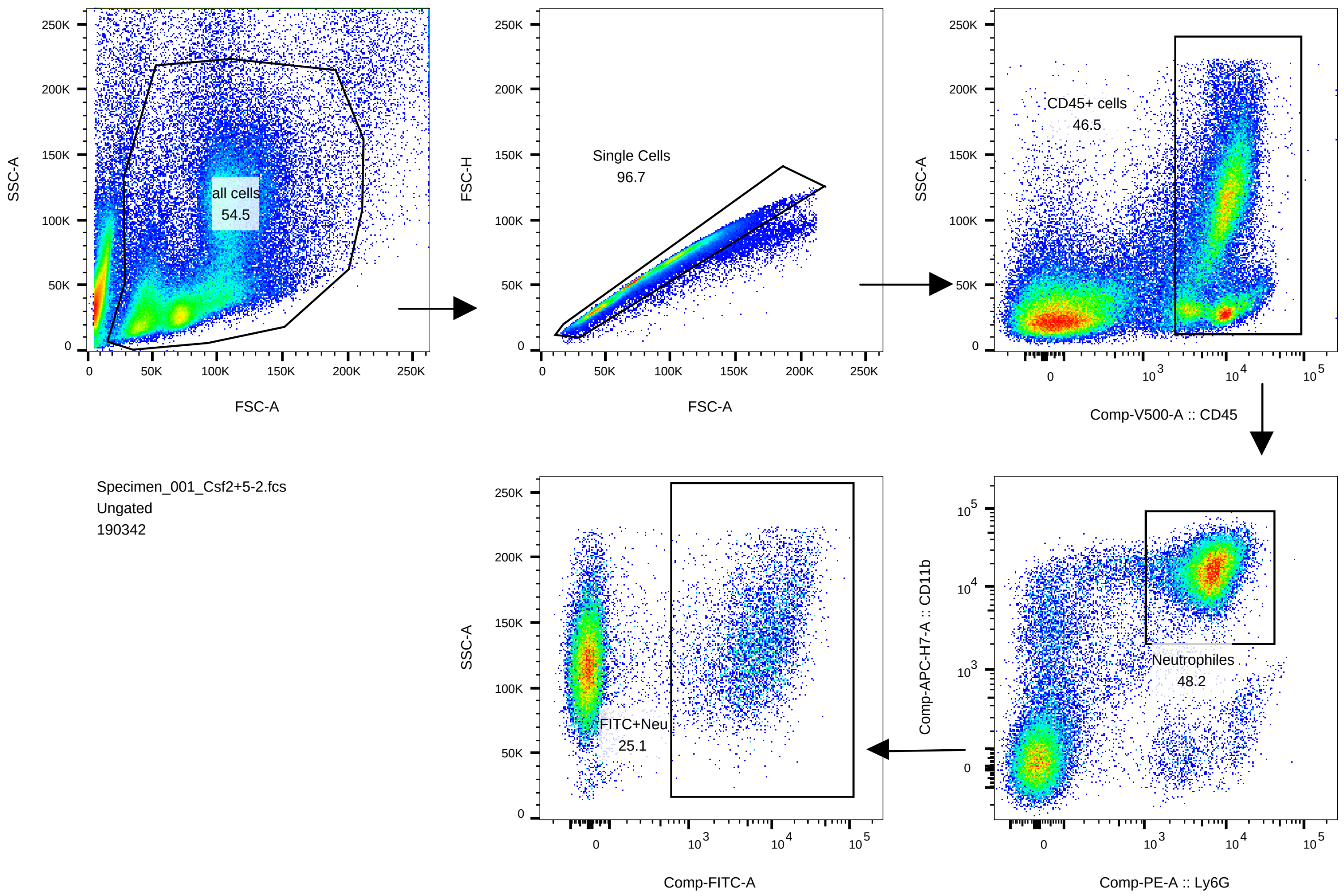
SSC-A
FSC-A
all cells
54.5
FSC-H
Single Cells
96.7
CD45+ cells
46.5
Comp-V500-A :: CD45
Specimen_001_Csf2+5-2.fcs
Ungated
190342
FITC+Neu
25.1
Comp-FITC-A
Comp-APC-H7-A :: CD11b
Neutrophiles
48.2
Comp-PE-A :: Ly6G

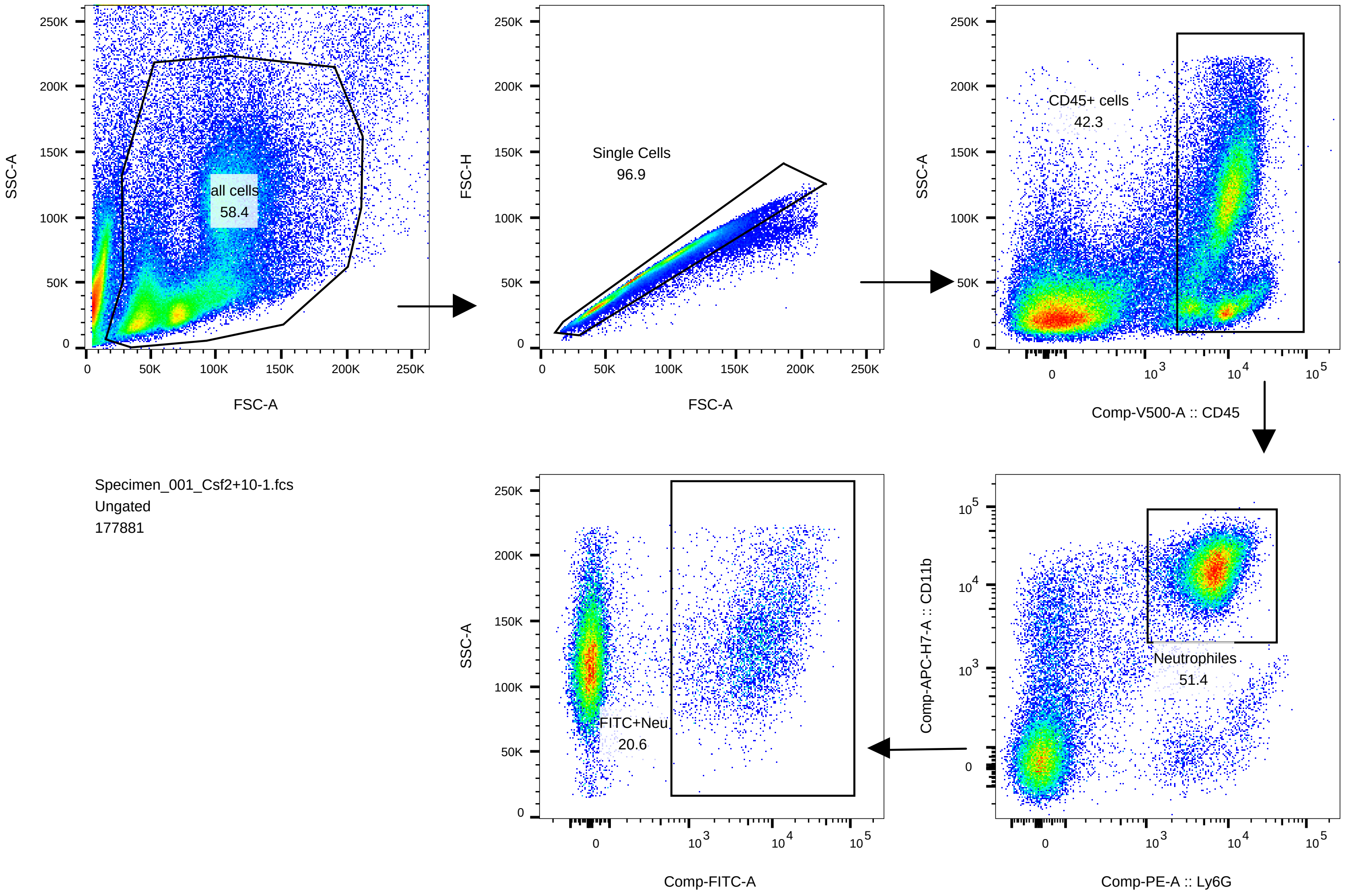

all cells
58.4
SSC-A
FSC-A
250K
200K
150K
100K
50K
0
Single Cells
96.9
FSC-H
CD45+ cells
42.3
Comp-V500-A :: CD45
Neutrophiles
51.4
Comp-APC-H7-A :: CD11b
Comp-PE-A :: Ly6G
FITC+Neu
20.6
Comp-FITC-A
Specimen_001_Csf2+10-1.fcs
Ungated
177881

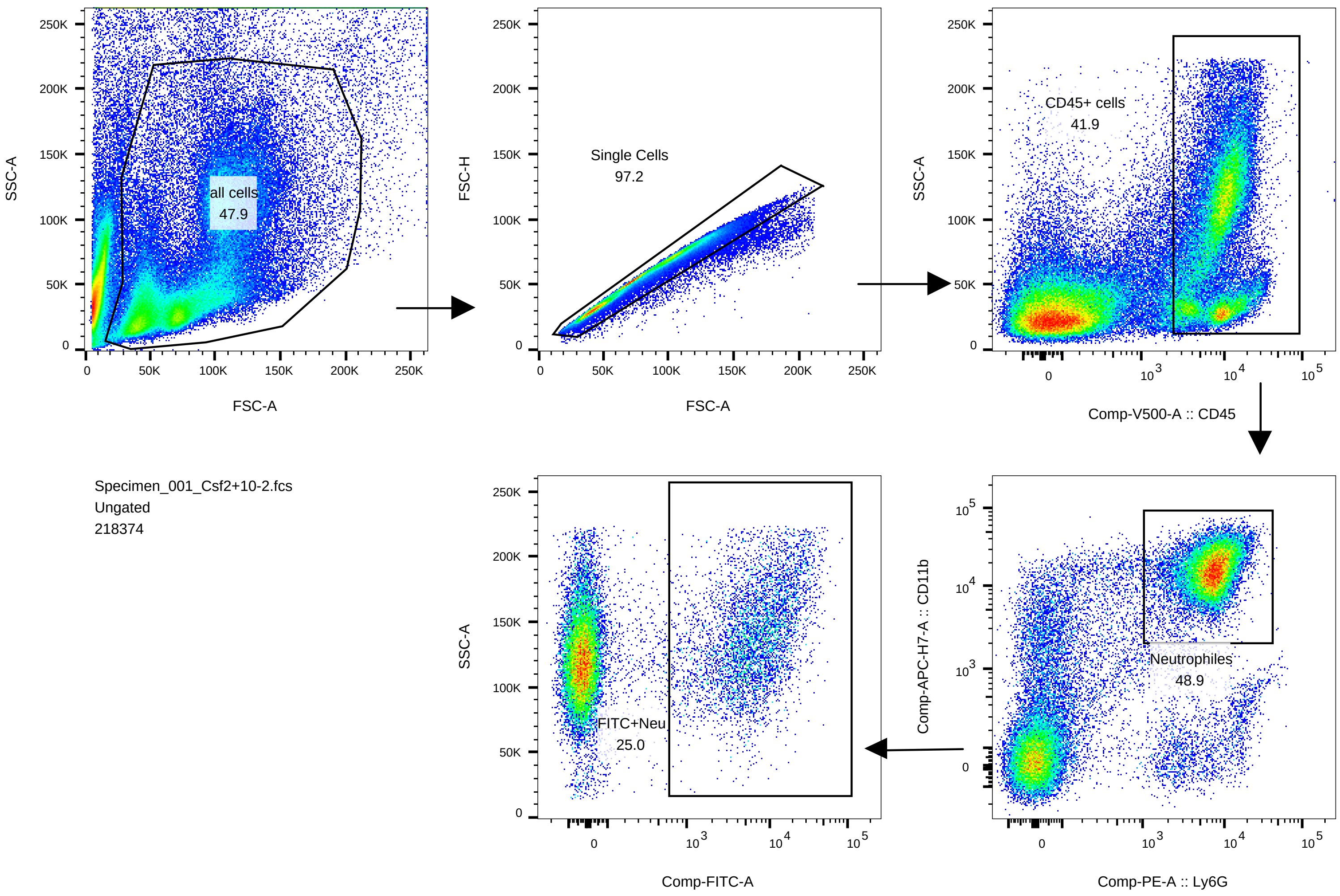
all cells
47.9
SSC-A
FSC-A
Single Cells
97.2
FSC-H
CD45+ cells
41.9
Comp-V500-A :: CD45
Specimen_001_Csf2+10-2.fcs
Ungated
218374
FITC+Neu
25.0
Comp-FITC-A
Neutrophiles
48.9
Comp-APC-H7-A :: CD11b
Comp-PE-A :: Ly6G

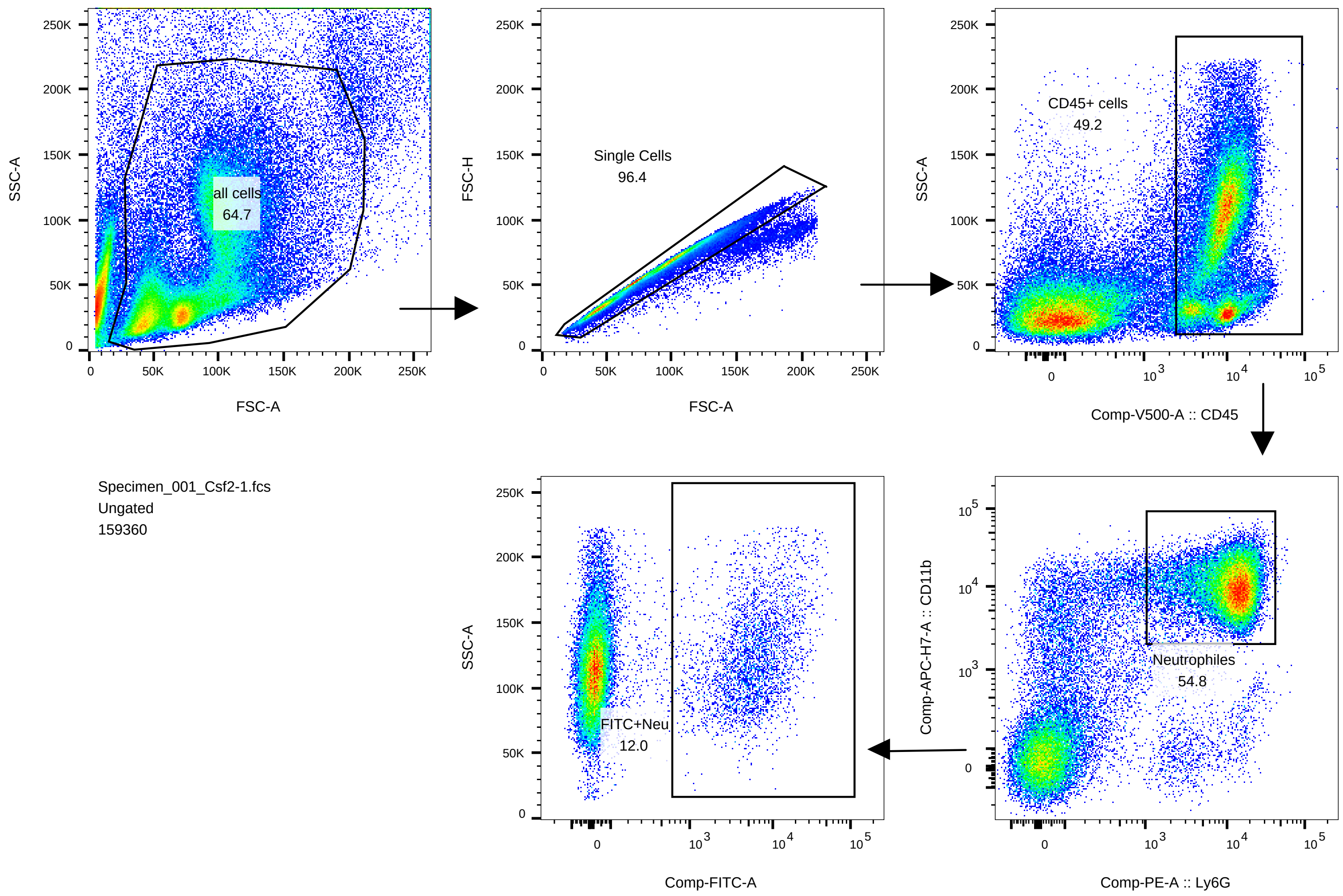

all cells
64.7
SSC-A
FSC-A
Single Cells
96.4
FSC-H
FSC-A
CD45+ cells
49.2
SSC-A
Comp-V500-A :: CD45
Specimen_001_Csf2-1.fcs
Ungated
159360
FITC+Neu
12.0
SSC-A
Comp-FITC-A
Neutrophiles
54.8
Comp-APC-H7-A :: CD11b
Comp-PE-A :: Ly6G

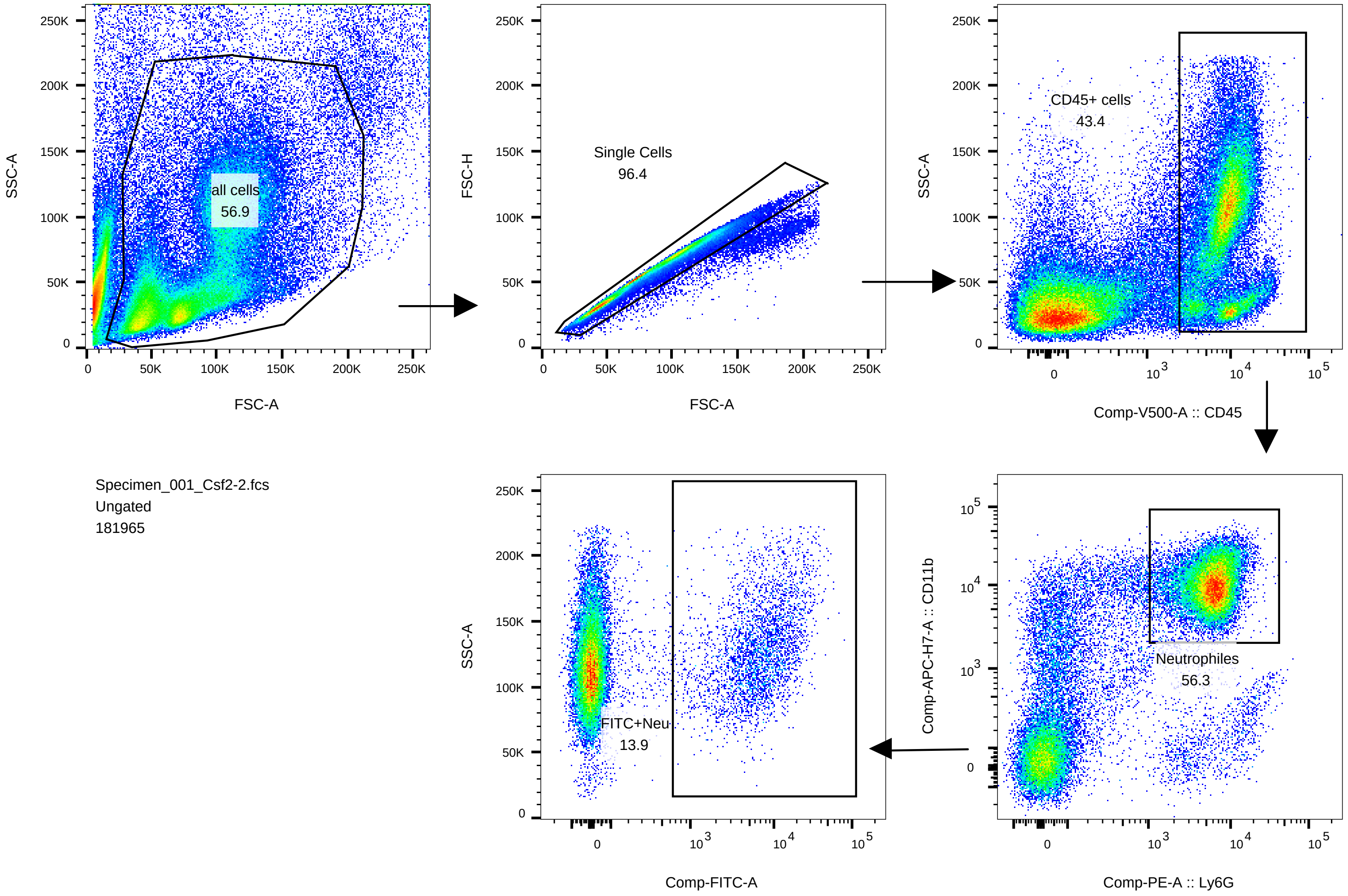

all cells
56.9
SSC-A
FSC-A
Single Cells
96.4
FSC-H
FSC-A
CD45+ cells
43.4
SSC-A
Comp-V500-A :: CD45
Specimen_001_Csf2-2.fcs
Ungated
181965
FITC+Neu
13.9
SSC-A
Comp-FITC-A
Neutrophiles
56.3
Comp-APC-H7-A :: CD11b
Comp-PE-A :: Ly6G

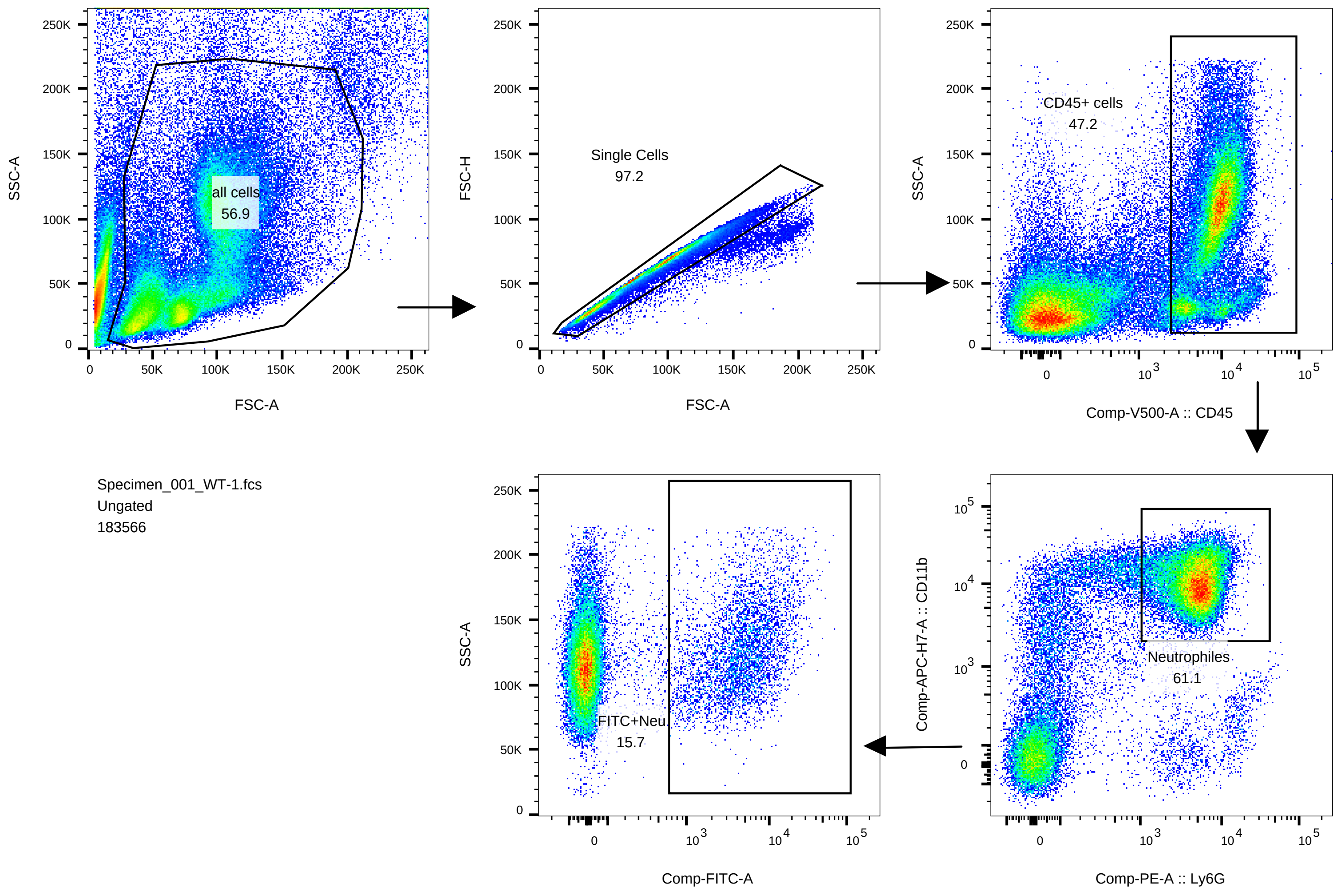
all cells
56.9
SSC-A
FSC-A
Single Cells
97.2
FSC-H
FSC-A
CD45+ cells
47.2
SSC-A
Comp-V500-A :: CD45
Specimen_001_WT-1.fcs
Ungated
183566
FITC+Neu
15.7
SSC-A
Comp-FITC-A
Neutrophiles
61.1
Comp-APC-H7-A :: CD11b
Comp-PE-A :: Ly6G

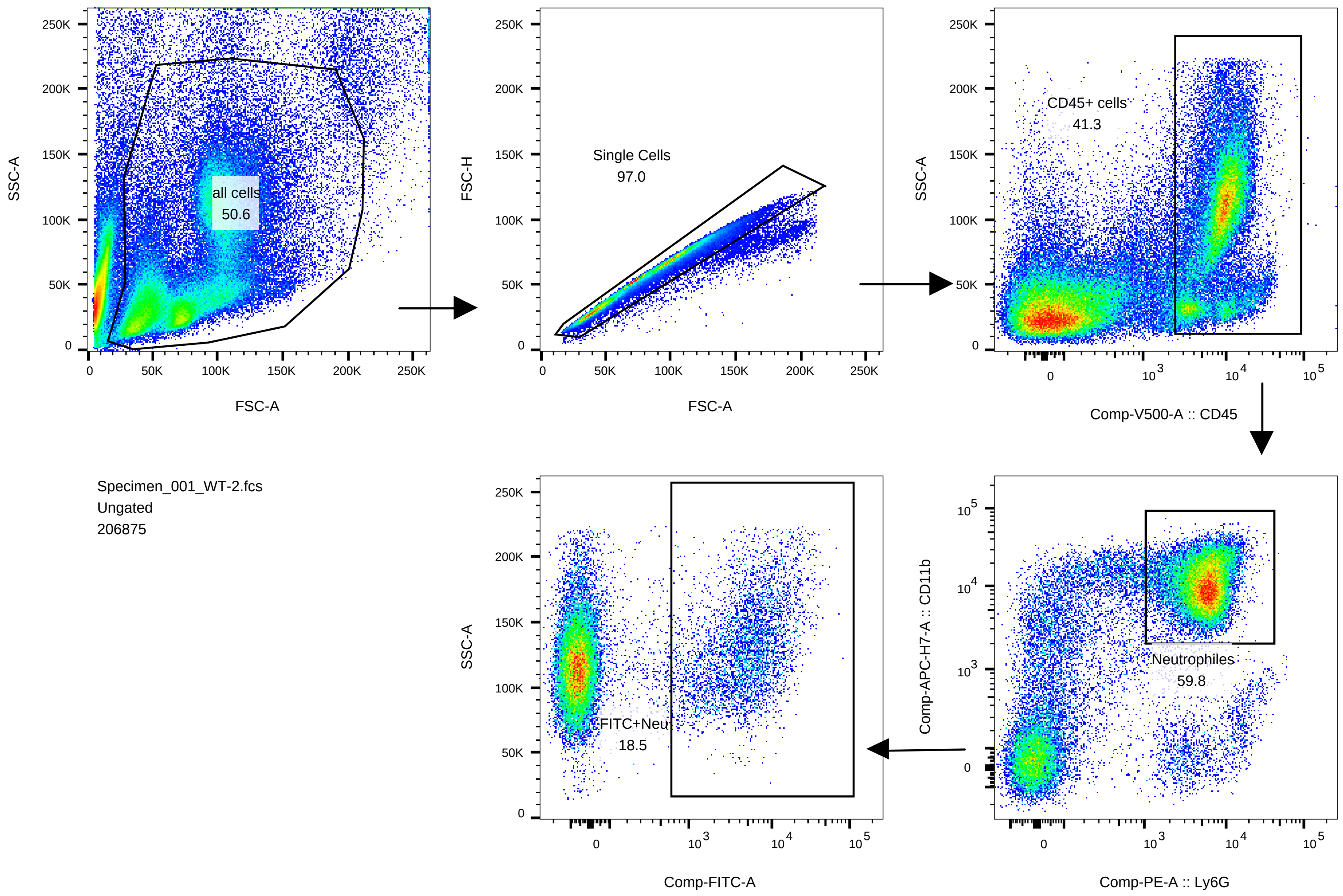

SSC-A
FSC-A
all cells
50.6
FSC-H
Single Cells
97.0
CD45+ cells
41.3
Comp-V500-A :: CD45
Specimen_001_WT-2.fcs
Ungated
206875
FITC+Neu
18.5
Comp-FITC-A
Comp-APC-H7-A :: CD11b
Neutrophiles
59.8
Comp-PE-A :: Ly6G
