# Supplementary material for: The power of GM-CSF: immune regulation in the defense against Phialophora verrucosa infection
Source: Front Immunol. 2025 Oct 20;16:1662183. doi: 10.3389/fimmu.2025.1662183 (PMC12580205; doi:10.3389/fimmu.2025.1662183)
Supplement: Supplementary file 5 [file DataSheet5.pdf]

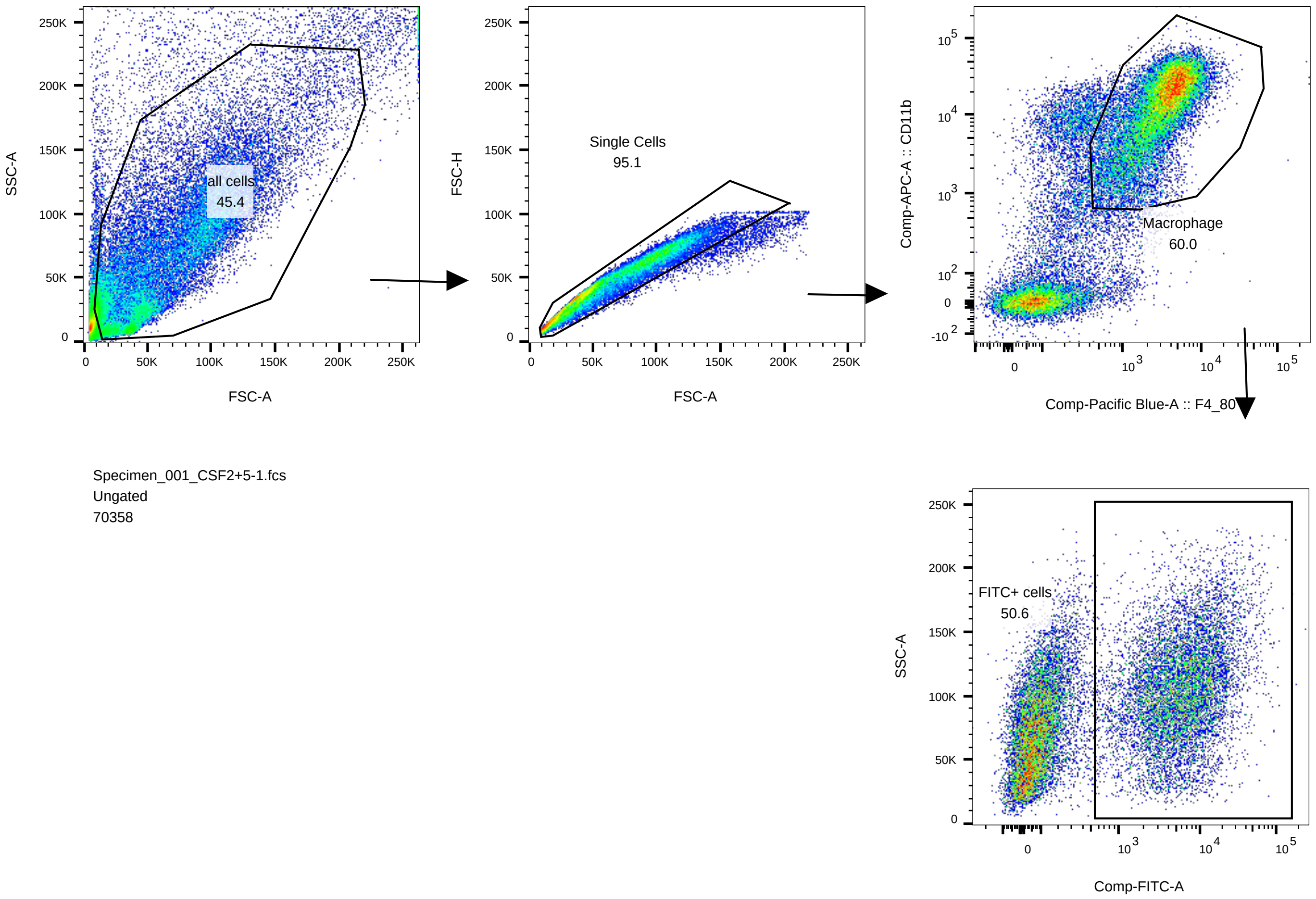
SSC-A
FSC-A
all cells
45.4
250K
200K
150K
100K
50K
0
FSC-H
Single Cells
95.1
Comp-APC-A :: CD11b
Comp-Pacific Blue-A :: F4_80
Macrophage
60.0
Comp-FITC-A
FITC+ cells
50.6
Specimen_001_CSF2+5-1.fcs
Ungated
70358

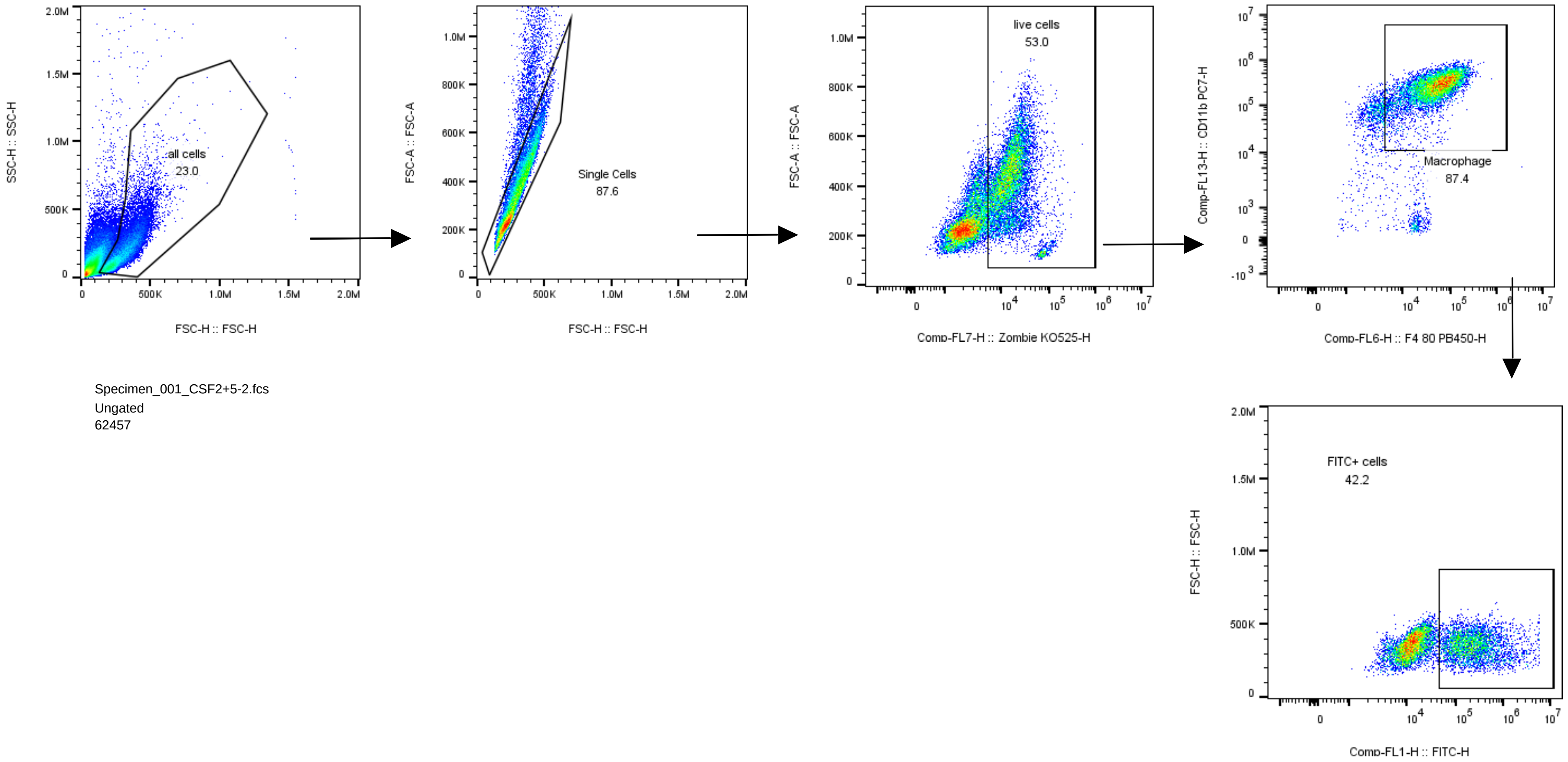
SSC-H :: SSC-H
FSC-H :: FSC-H
all cells
23.0
Specimen_001_CSF2+5-2.fcs
Ungated
62457
FSC-A :: FSC-A
FSC-H :: FSC-H
Single Cells
87.6
FSC-A :: FSC-A
Comp-FL7-H :: Zombie KO525-H
live cells
53.0
Comp-FL13-H :: CD11b PC7-H
Comp-FL6-H :: F4 80 PB450-H
Macrophage
87.4
FSC-H :: FSC-H
Comp-FL1-H :: FITC-H
FITC+ cells
42.2

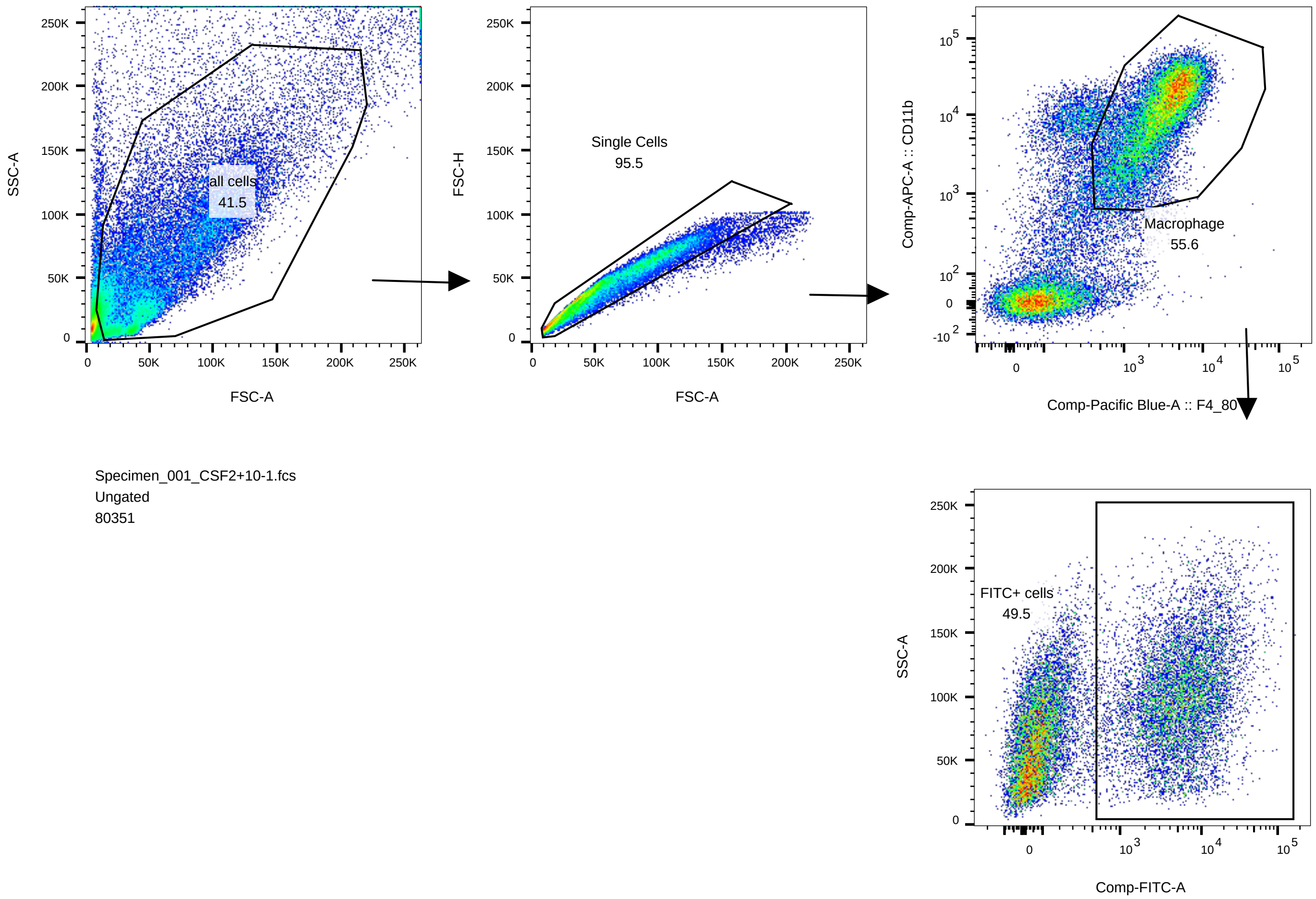
all cells
41.5
SSC-A
FSC-A
Single Cells
95.5
FSC-H
FSC-A
Macrophage
55.6
Comp-APC-A :: CD11b
Comp-Pacific Blue-A :: F4_80
Specimen_001_CSF2+10-1.fcs
Ungated
80351
FITC+ cells
49.5
SSC-A
Comp-FITC-A

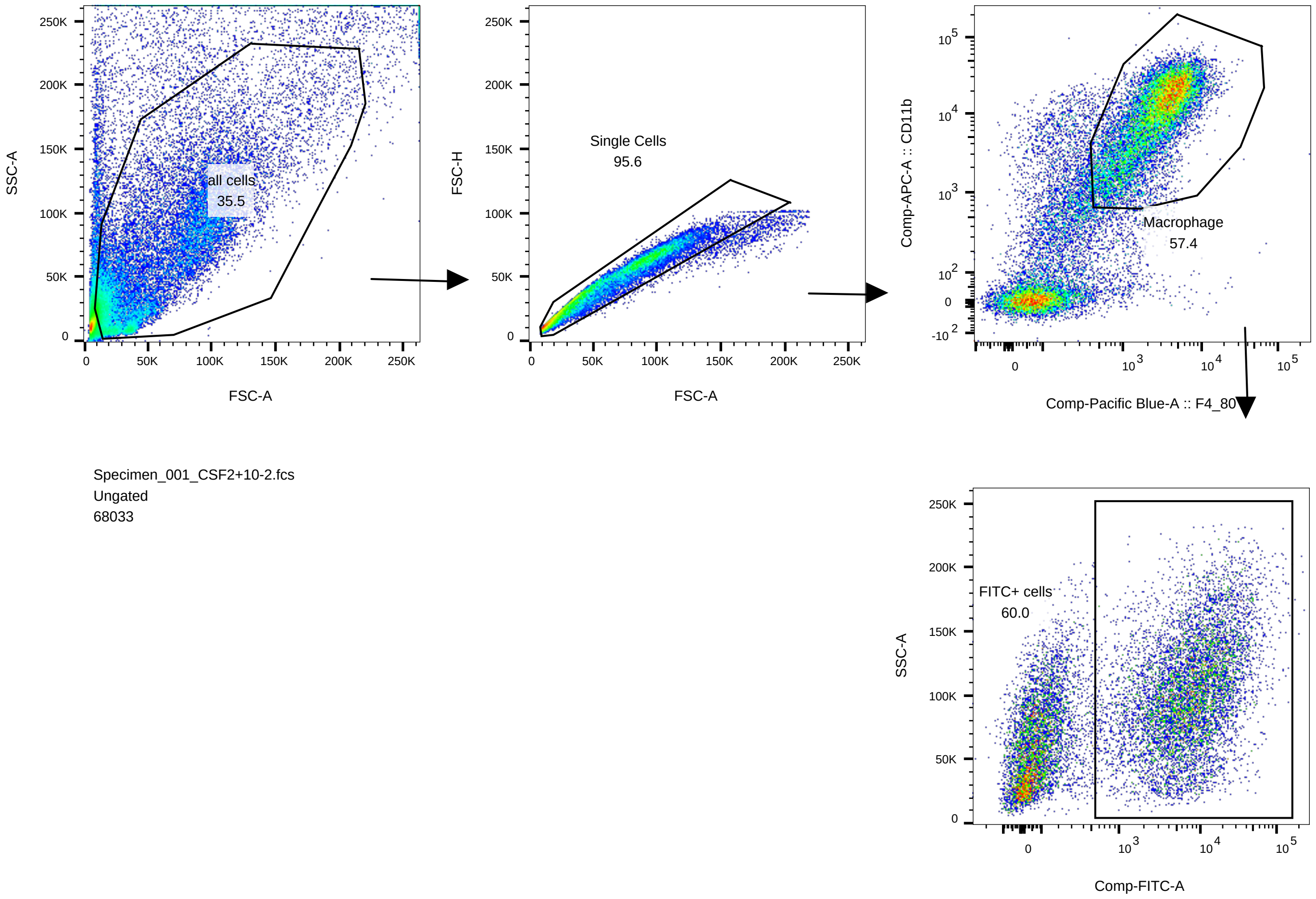

SSC-A
FSC-A
all cells
35.5
FSC-H
FSC-A
Single Cells
95.6
Comp-APC-A :: CD11b
Comp-Pacific Blue-A :: F4_80
Macrophage
57.4
SSC-A
Comp-FITC-A
FITC+ cells
60.0
Specimen_001_CSF2+10-2.fcs
Ungated
68033

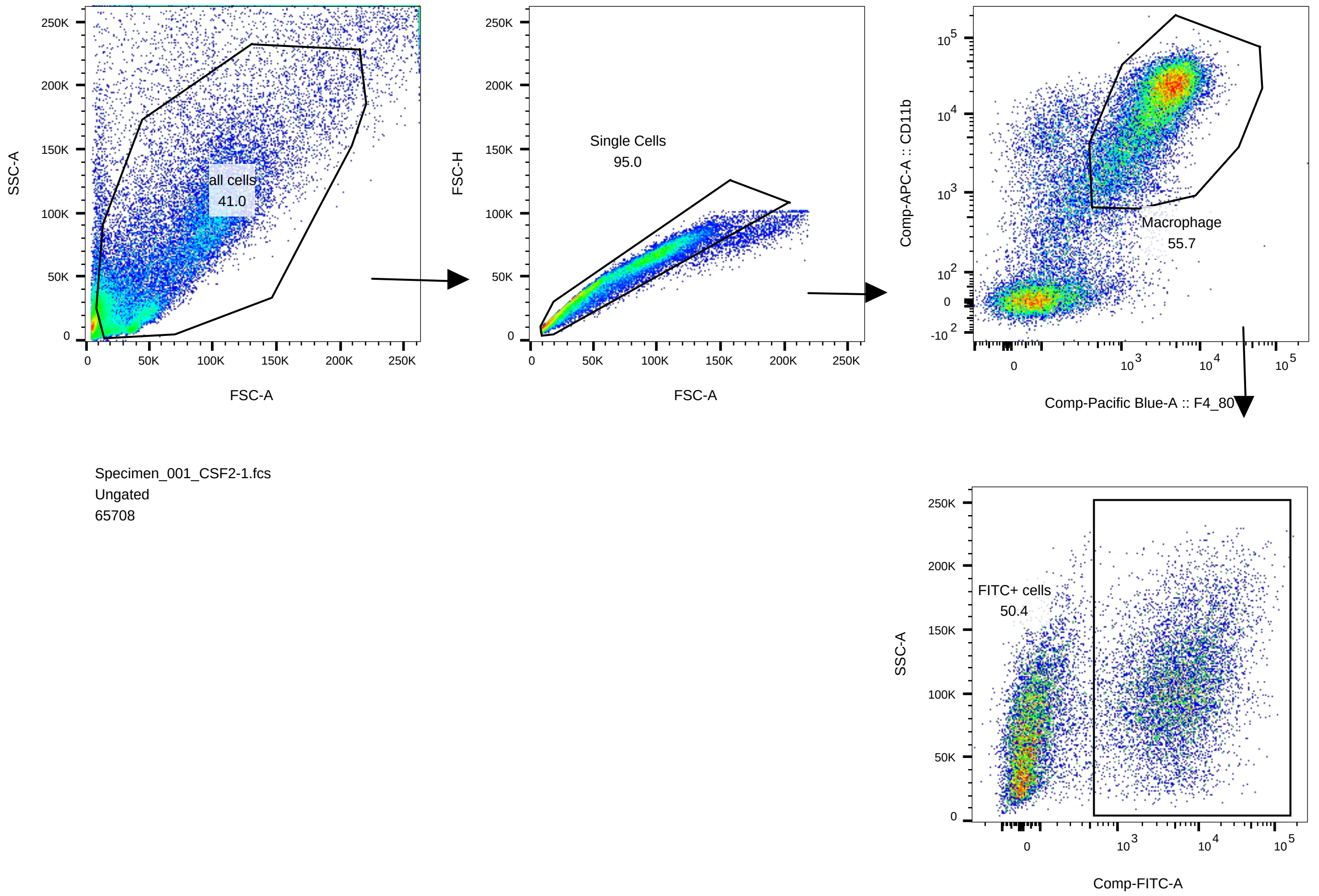
SSC-A
FSC-A
all cells
41.0
FSC-H
FSC-A
Single Cells
95.0
Comp-APC-A :: CD11b
Comp-Pacific Blue-A :: F4_80
Macrophage
55.7
Specimen_001_CSF2-1.fcs
Ungated
65708
SSC-A
Comp-FITC-A
FITC+ cells
50.4

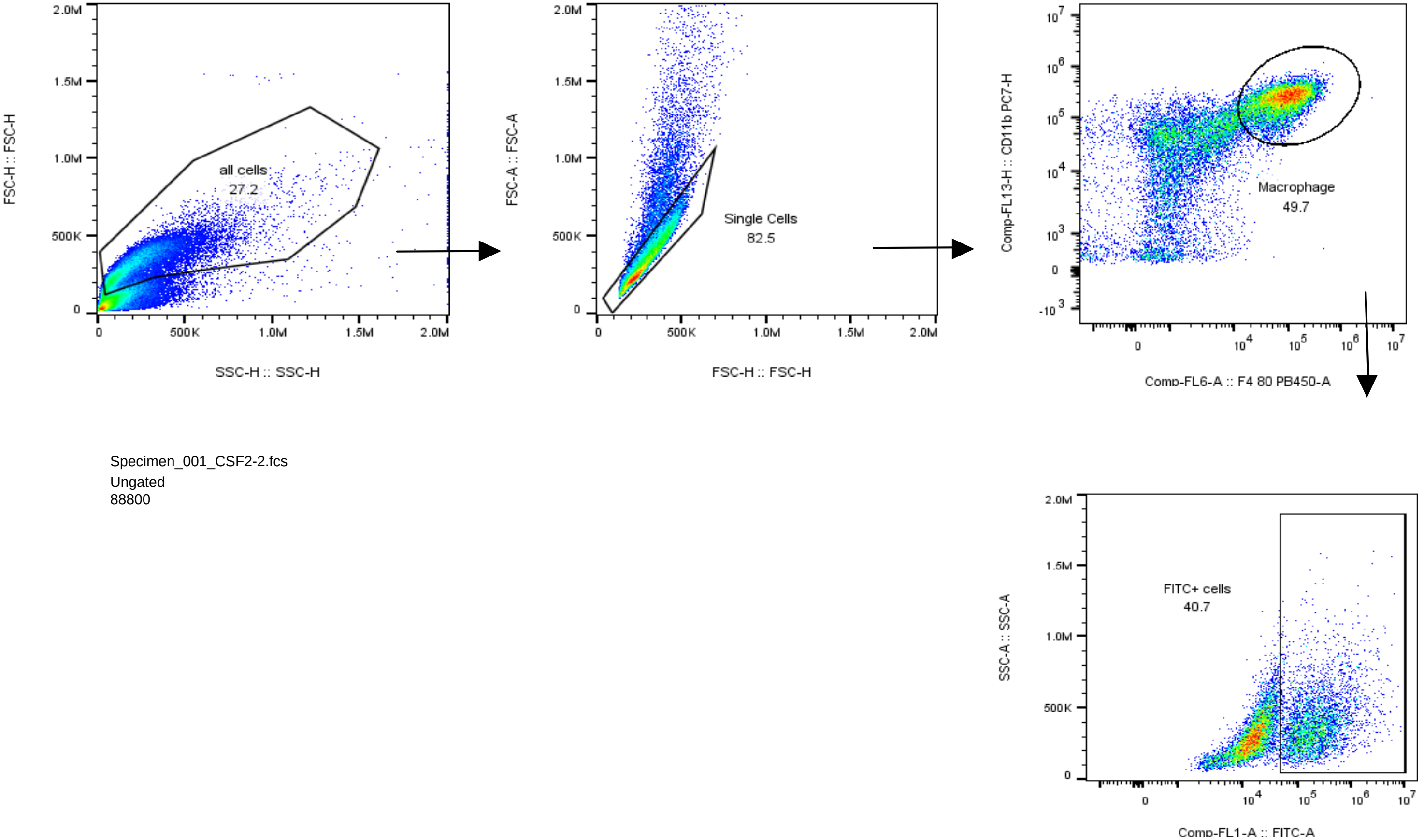

Specimen_001_CSF2-2.fcs
Ungated
88800

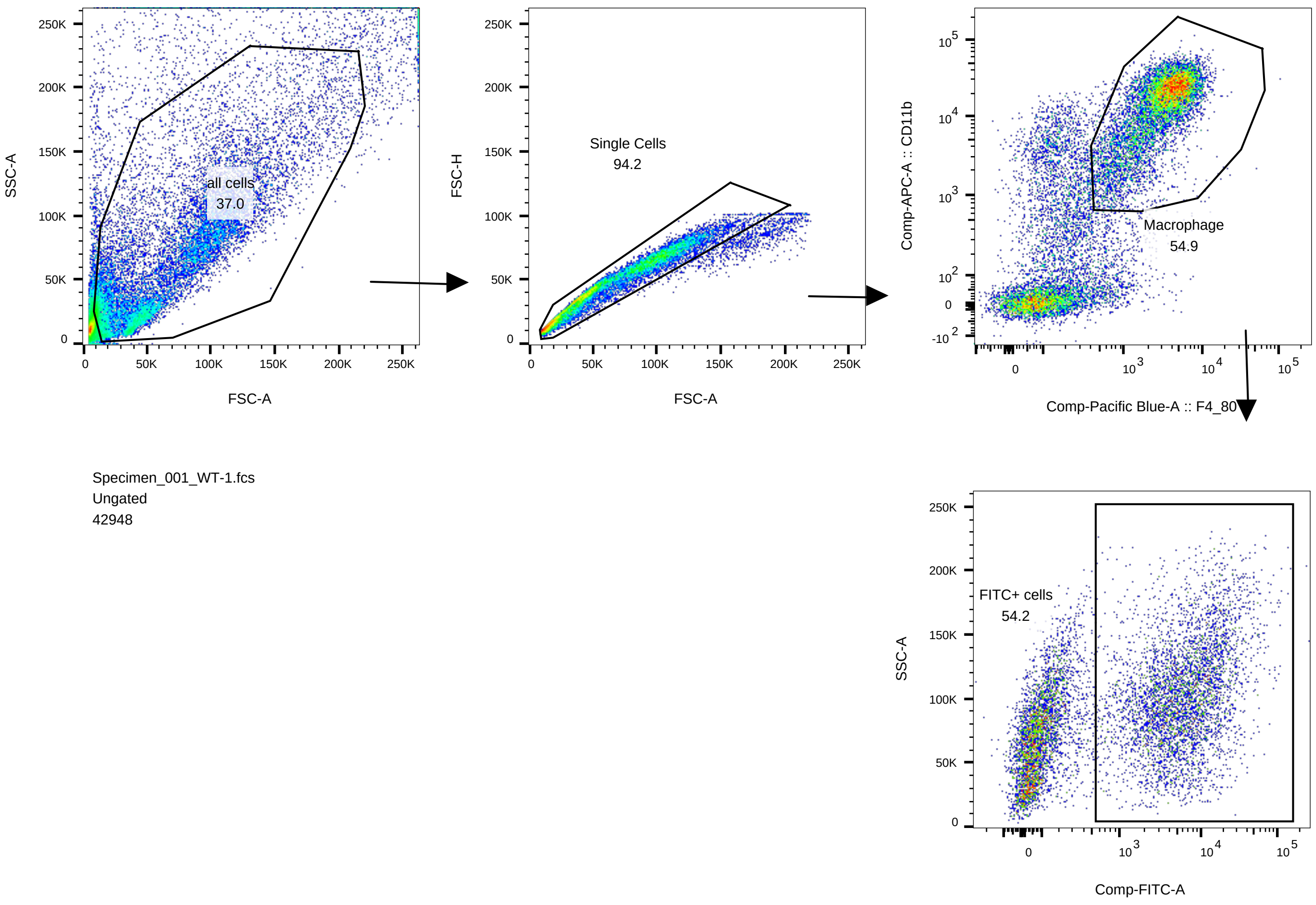

SSC-A
250K
200K
150K
100K
50K
0
0
50K
100K
150K
200K
250K
FSC-A
all cells
37.0
FSC-H
Single Cells
94.2
FSC-A
Comp-APC-A :: CD11b
Macrophage
54.9
Comp-Pacific Blue-A :: F4_80
SSC-A
FITC+ cells
54.2
Comp-FITC-A
Specimen_001_WT-1.fcs
Ungated
42948

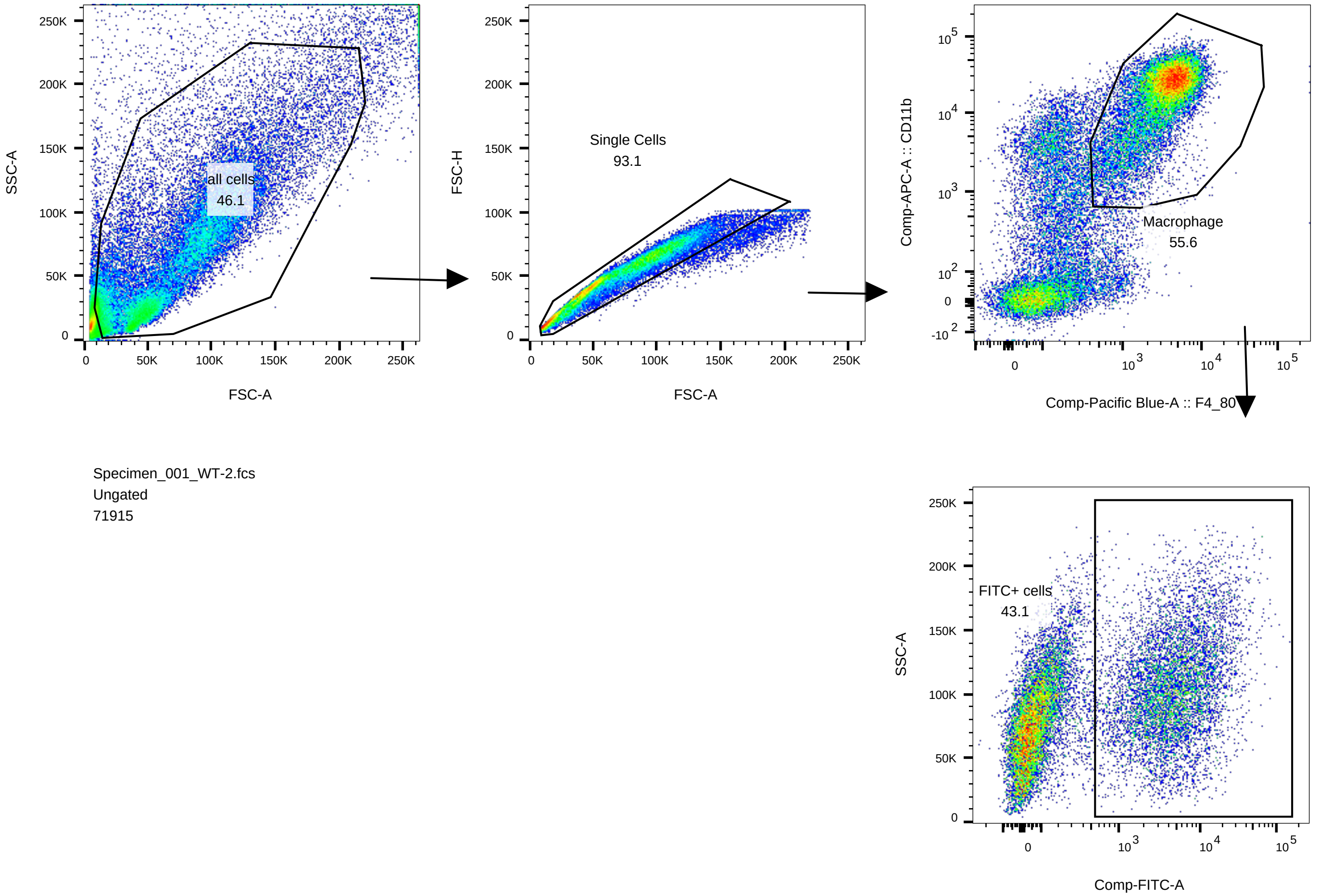
all cells
46.1
SSC-A
FSC-A
0
50K
100K
150K
200K
250K
Specimen_001_WT-2.fcs
Ungated
71915
Single Cells
93.1
FSC-H
FSC-A
Macrophage
55.6
Comp-APC-A :: CD11b
Comp-Pacific Blue-A :: F4_80
FITC+ cells
43.1
SSC-A
Comp-FITC-A
